# Whole-Exome Sequencing in the Isolated Populations of Cilento from South Italy

**DOI:** 10.1038/s41598-019-41022-6

**Published:** 2019-03-11

**Authors:** T. Nutile, D. Ruggiero, A. F. Herzig, A. Tirozzi, S. Nappo, R. Sorice, F. Marangio, C. Bellenguez, A. L. Leutenegger, M. Ciullo

**Affiliations:** 1 Institute of Genetics and Biophysics A. Buzzati-Traverso-CNR, Naples, Italy; 2 IRCCS Neuromed, Pozzilli, Isernia Italy; 3 Inserm, UMR 946, Genetic variation and Human diseases, F-75010 Paris, France; 4 Université Paris-Diderot, Sorbonne Paris Cité, UMR946, F-75010 Paris, France; 5 AORN Santobono-Pausilipon Hospital, Naples, Italy; 6 Inserm, U1167, RID-AGE-Risk factors and molecular determinants of aging-related diseases, F-59000 Lille, France; 7 Institut Pasteur de Lille, F-59000 Lille, France; 8 Univ. Lille, U1167-Excellence Laboratory LabEx DISTALZ, F-59000 Lille, France

## Abstract

The present study describes the genetic architecture of the isolated populations of Cilento, through the analysis of exome sequence data of 245 representative individuals of these populations. By annotating the exome variants and cataloguing them according to their frequency and functional effects, we identified 347,684 variants, 67.4% of which are rare and low frequency variants, and 1% of them (corresponding to 319 variants per person) are classified as high functional impact variants; also, 39,946 (11.5% of the total) are novel variants, for which we determined a significant enrichment for deleterious effects. By comparing the allele frequencies in Cilento with those from the Tuscan population from the 1000 Genomes Project Phase 3, we highlighted an increase in allele frequency in Cilento especially for variants which map to genes involved in extracellular matrix formation and organization. Furthermore, among the variants showing increased frequency we identified several known rare disease-causing variants. By different population genetics analyses, we corroborated the status of the Cilento populations as genetic isolates. Finally, we showed that exome data of Cilento represents a useful local reference panel capable of improving the accuracy of genetic imputation, thus adding power to genetic studies of human traits in these populations.

## Introduction

Genomic studies are playing a crucial role in enlarging knowledge of genetic variation between and within populations^1^ and in identifying the genetic bases of complex traits/diseases. In the last few years, a huge amount of genetic data have been collected by whole-exome sequencing (WES) and whole-genome sequencing (WGS) studies^2–10^, performed on different general and isolated populations worldwide, including isolates from Northern Italy^11^. These studies revealed an enrichment for rare and low frequency variants, and identified variants private to each examined population. However, none of these studies has included populations from Southern Italy, leading to an underrepresentation of their genetic diversity in the genomic data collected to date. To try to fill this gap, we have performed a whole-exome sequencing study on individuals from the populations of three small villages (Campora, Gioi, and Cardile) located in an inland area of the “National Park of Cilento and Vallo di Diano”, in Southern Italy, included in the “Genetic Park of Cilento and Vallo di Diano Project”. Campora and Gioi were settled at the beginning of the 11^th^ century by Greek and Byzantine monks, while Cardile was founded much later, in the mid-18th century, by a group of individuals coming from Gioi. Afterwards these populations suffered severe bottlenecks due to famine and the epidemic of bubonic plague in the 17^th^ century. Then, they were affected by different strong waves of emigration that drastically reduced the population size and experienced a high level of reproductive isolation until mid-20th century. As a consequence of the high percentage of endogamous marriages among their inhabitants, in each isolate the majority of the current population are connected in a unique huge genealogy, and descend from a few number of founding lineages. Also, the presence of inbreeding and a higher level of linkage disequilibrium characterize the Cilento isolates when compared to outbred populations^12,13^. Data of the Cilento isolates have contributed to many Genome-wide association studies (GWAS) for several complex traits of clinical interest^14–20^.

In this work we used the data coming from the WES study performed on the Cilento isolates to describe their genetic architecture, annotating the identified variants, cataloguing them according to their frequency and functional effects, and comparing their frequencies with those from a general Italian population. Because of the initial population bottleneck followed by genetic drift during the first generations, it is expected that in these isolates the frequencies of some genetic variants may have changed compared to those of the founding pool. Some variants probably disappeared, thus limiting heterogeneity, while others may have become more frequent, which could facilitate their identification. Overall, changes in allele frequency are larger for rare variants than common ones. Thus, allele frequencies in isolated populations and large populations are expected to differ the most at rare variants. Therefore, rare variants with increased allele frequencies in isolated populations could be more easily identified in genetic studies of traits with a Mendelian or complex inheritance. Thus, we focused our attention on variants which have an increased allele frequency in the Cilento isolates compared to a general Italian population. Among those, we searched for variants of particular interest, such as those having a functional impact on rare diseases. Furthermore, we performed different population genetics analyses to better characterize these populations as genetic isolates. Finally, we tested the utility of using exome data as a local reference panel to improve the accuracy of imputation and potentially increase the power of further genetic studies on human traits in these populations.

## Results

### Description of Cilento exome sequencing

The analysis of the whole-exome data on the Cilento populations revealed high-performance sequencing, with an aligned read mean depth on target regions of 75X.

**Figure 1:**
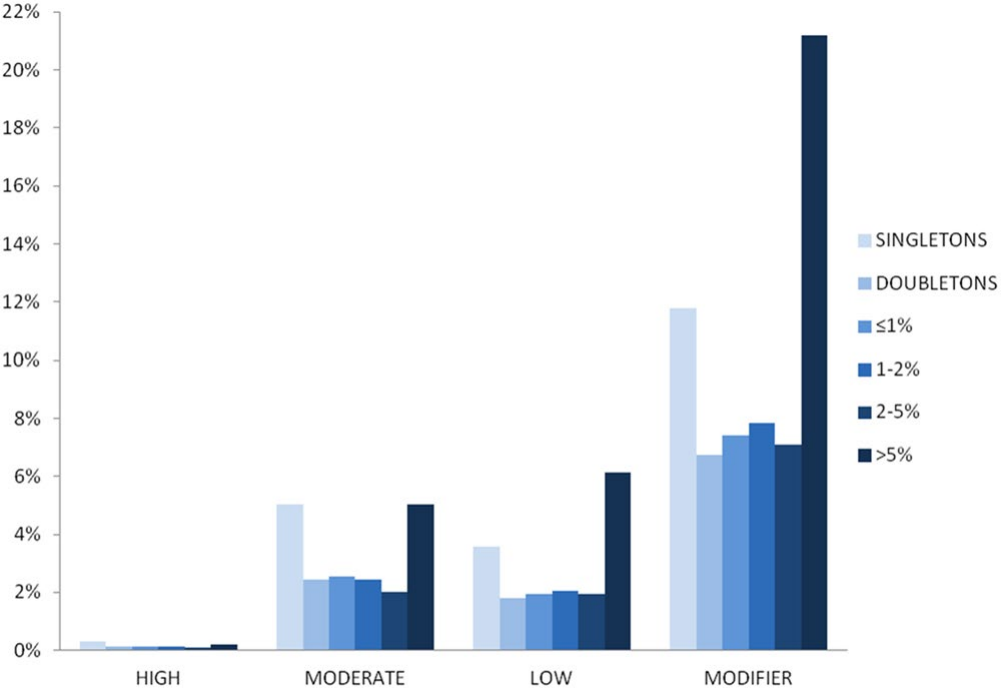
Cilento variants. The percentage of variants found in the Cilento whole-exome sequencing study, categorized by functional impact and minor allele frequency.

We found 347,684 variants, 43.8% of which are rare (Minor Allele Frequency, MAF ≤ 1%) and 23.6% have MAF between 1% and 5%, giving a total of 67.4% having low frequency (MAF ≤ 5%). 20.7% of all variants identified in Cilento are singletons (Minor Allele Count, MAC = 1). We grouped sequence variants into four functional impact categories, in order of decreasing severity: 1) HIGH (Loss of Function), including stop-gain or -loss variants, frameshift variants, splice donor or acceptor variants and initiator codon variants; 2) MODERATE, including missense variants and in-frame insertions and deletions; 3) LOW, including synonymous variants, stop retained variants, incomplete terminal codon variants and splice-region variants; and 4) MODIFIER, including intronic and intergenic variants, 5′ and 3′ UTR variants, regulatory region and transcription factor binding site (TFBS) variants, miRNA variants and non-coding exon variants. We identified 3,562 HIGH impact variants, representing approximately 1% of all the variants. On average, each individual carries 319 (range 279–365) HIGH impact variants, of which 50 (range 31–68) are in a homozygous state. Rare variants are the most represented in the HIGH impact category (58.6%), while frequent variants (MAF > 1%) are more prevalent in both the LOW and MODIFIER categories (58.1% and 58.2% respectively) (Fig. 1 and Supplementary Table 1).

### Cilento novel variants

**Figure 2:**
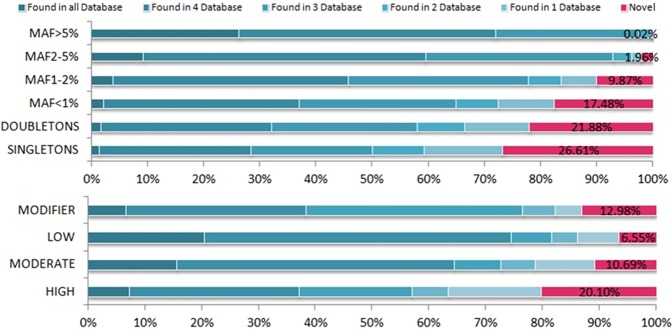
Cilento novel variants. Percentage of Cilento novel variants (in pink) and shared variants (in blue) according to Minor Allele Frequency category and functional impact. The shared variants are grouped according to the number of reference databases in which they were found (indicated by different blue shades).

Comparing the Cilento exome data with five largely used reference datasets (see Methods), we found that 39,946 variants, corresponding to 11.5% of all the identified variants, are novel, while 88.5% were found in at least one reference database and 10.9% were shared with all the five databases (Supplementary Table 2). The percentage of novel variants in each frequency class decreases as MAF increases (as presented in Fig. 2 and in Supplementary Table 2). In accordance with the capacity of WES in identifying new rare variants^2,10^, 87.3% of Cilento novel variants have a MAF below 1% while the remaining 12.7% is distributed in the MAF classes above 1%. However, we also identified 19 novel variants (0.047%) with a MAF > 5%.

**Figure 3:**
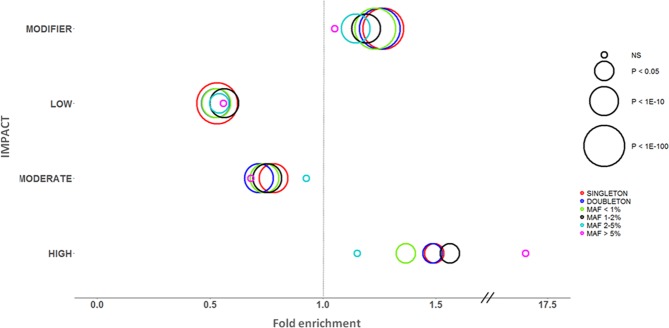
Functional enrichment of Cilento novel variants. The analysis was performed comparing novel variants with those shared with at least one reference database. The size of the circles represents the significance level of the two-sided test based on asymptotic normal distribution (Fisher exact test for the following categories: HIGH/MAF > 5%, MODERATE/MAF > 5%, and LOW/MAF > 5%). The x-axis indicates the fold enrichment, the vertical line indicates no enrichment (the proportions in the NOVEL and the SHARED set are equal). NS = not significant.

According to their impact, 70.2% of the novel variants were included in the MODIFIER class, 9.9% in the LOW, 18.1% in the MODERATE, and 1.8% (716 variants) in the HIGH class (Supplementary Table 2). We observed a statistical significant enrichment of Cilento novel variants in the HIGH and MODIFIER categories and a depletion in the MODERATE and LOW classes compared to variants shared with the other databases (Supplementary Table 3 and Fig. 3). Overall, these differences were more significant for those variants belonging to the lower MAF classes. In detail, for all variants with MAF < 2% we observed a significant difference in proportion in all impact classes, while for variants with MAF 2–5% we observed only a significant enrichment and depletion in the MODIFIER and the LOW classes respectively.

### Exome variants showing an increase in allele frequency in Cilento

To estimate the genetic drift in the Cilento isolates, we compared variant allele frequencies (AF) in Campora, Gioi and Cardile with those of the Tuscans (TSI) from 1000 Genomes Phase 3 v5 (1KG_Ph3)^1^, a general Italian population. We will use the term ‘increased allele frequency variants’ to describe variants with an AF that is higher in Cilento isolates than in TSI. Looking at the variants shared by each Cilento isolate with the Tuscany population (see Methods), we found that 118,795 (58.0%), 130,569 (59.8%), and 107,512 (57.4%) variants showed increased AF in Campora, Gioi, and Cardile, respectively, with respect to TSI (Supplementary Table 4). Among these, 93,309 increased allele frequency variants in Campora, 97,439 in Gioi, and 87,309 in Cardile were not monomorphic in TSI, and for these polymorphic variants we calculated the fold increase in AF respect to TSI following the same approach used in a work on two Greek population isolates^21^. Although the vast majority of these variants exhibits a fold increase <5, for 5% of variants in Campora, 3.7% in Gioi, and 6.8% in Cardile we observed a fold increase >5. In particular, 30 variants in Campora, 7 in Gioi, and 35 in Cardile showed more than 25-fold increase in AF compared to TSI.

The majority of increased allele frequency variants show, as expected, a MAF > 5% in all the three villages (53.9% in Campora, 46.5% in Gioi and 60.7% in Cardile), but a considerable proportion of them (13.1%, 18.4% and 14.5% in Campora, Gioi and Cardile respectively), which correspond to singletons or doubletons in TSI, are still rare (MAF < 1%) in Cilento villages.

According to their functional impact, 0.8% of variants showing an increase in AF in each of the three populations (898 in Campora, 977 in Gioi, and 826 in Cardile) were included in the HIGH category, although the majority of increased allele frequency variants were in the MODIFIER class (64.0% in Campora, 63.2% in Gioi, and 63.6% in Cardile).

Conversely, 85,879 (42.0% of all variants) variants in Campora, 87,701 (40.2%) in Gioi, and 79,747 (42.6%) in Cardile had decreased AF compared with TSI.

### Gene-enrichment analysis

To discern whether the genes containing the variants showing AF increases in each isolate are located in specific pathways, an over-representation analysis was performed using the ConsensusPathDB (CPDB) program^22^. Because of the small sample size of the Cilento isolates (Cardile in particular), we only considered variants with a fold increase in frequency >5 respect to TSI or variants monomorphic in TSI that have a MAF ≥ 0.0223 in Cilento villages (corresponding to a fold increase of 5 for a singleton in TSI). This MAF threshold ensures that variants analysed have at least MAC = 3 in Cardile and MAC = 5 in Gioi and Campora.

According to these criteria, 9,126 variants in Campora, 6,882 in Gioi and 11,512 in Cardile (only 354 in common between the three sets), were used for the analysis. These variants mapped to 8,442, 6,710, and 9,979 genes respectively (of which 2,087 are in common). Out of these genes, 4,087 in Campora, 3,292 in Gioi, and 4,766 in Cardile were found in at least one pathway.

The over-representation analysis identified 23 significantly enriched pathways in Campora, 69 in Gioi, and 73 in Cardile (Supplementary Table 5). We consider pathways to be significant if they have a q-value (multiple-testing adjusted p-value) < 0.05. Of these, 13 pathways were found enriched in all the three analyses, suggesting that, despite the very low number of increased allele frequency variants in common between the three villages, many are located in genes belonging to the same pathways. Furthermore, additional pathways were found in common between two villages: 13 were found both in Gioi and Cardile, 3 in Campora and Cardile and 1 in Campora and Gioi. (Table 1). Interestingly, the majority the pathways significantly enriched in common among the three villages (8 out of 13) are related to the extracellular matrix (ECM) formation and organization - in particular to the collagen component - and to its interaction with cell receptors. The remaining enriched pathways are related to Vesicle-mediated transport and Transmembrane transport, Protein digestion and absorption, Axon guidance, and Stimuli-sensing channels.

**Table 1:** List of the 13 over-represented pathways in common between the ConsensusPathDB analyses performed on the three villages.

Pathway name	Pathway size	Campora			Gioi			Cardile			Genes contained in common	Increased variants in common
		Genes contained	p-value	q-value	Genes contained	p-value	q-value	Genes contained	p-value	q-value		
Axon guidance	357	154 (43.3%)	2.53E-06	1.36E-03	126 (35.4%)	1.97E-05	5.97E-03	171 (48.0%)	1.09E-05	2.37E-03	53	18
Beta1 integrin cell surface interactions	66	36 (54.5%)	1.02E-04	2.14E-02	32 (48.5%)	4.98E-05	8.98E-03	40 (60.6%)	8.20E-05	1.01E-02	13	3
Collagen biosynthesis and modifying enzymes	67	42 (62.7%)	1.78E-07	1.39E-04	31 (46.3%)	1.94E-04	1.75E-02	41 (61.2%)	4.88E-05	7.64E-03	20	5
Collagen chain trimerization	44	33 (75.0%)	4.57E-09	1.73E-05	27 (61.4%)	5.55E-07	5.01E-04	33 (75.0%)	3.20E-07	1.25E-04	20	5
Collagen formation	91	53 (58.2%)	1.56E-07	1.39E-04	40 (44.0%)	1.02E-04	1.22E-02	59 (64.8%)	6.27E-08	3.06E-05	26	5
ECM-receptor interaction - Homo sapiens	82	44 (53.7%)	3.10E-05	8.36E-03	43 (52.4%)	1.70E-07	2.05E-04	54 (65.9%)	1.04E-07	4.50E-05	20	5
Extracellular matrix organization	293	136 (46.4%)	8.15E-08	1.39E-04	118 (40.3%)	1.81E-08	6.55E-05	165 (56.3%)	9.41E-12	3.68E-08	62	9
Focal adhesion - Homo sapiens	199	91 (45.7%)	2.27E-05	6.58E-03	78 (39.2%)	1.48E-05	5.92E-03	105 (52.8%)	3.78E-06	9.86E-04	33	9
Integrin	124	62 (50.0%)	1.67E-05	5.36E-03	59 (47.6%)	9.41E-08	1.70E-04	77 (62.1%)	1.13E-08	1.11E-05	35	7
Protein digestion and absorption - Homo sapiens	90	48 (53.3%)	1.70E-05	5.36E-03	41 (45.6%)	3.05E-05	6.94E-03	50 (55.6%)	2.54E-04	2.24E-02	24	6
Stimuli-sensing channels	102	56 (54.9%)	1.03E-06	6.49E-04	41 (40.2%)	8.21E-04	4.36E-02	60 (58.8%)	5.92E-06	1.45E-03	24	6
Transport of small molecules	666	265 (39.9%)	3.17E-06	1.50E-03	206 (31.0%)	6.83E-04	3.74E-02	324 (48.8%)	1.38E-10	2.69E-07	86	19
Vesicle-mediated transport	620	237 (38.2%)	2.73E-04	4.29E-02	205 (33.1%)	1.18E-05	5.92E-03	295 (47.6%)	2.38E-08	1.55E-05	74	22

p-values are calculated according to the hypergeometric test based on the number of genes present in both the pathway-based set and input list of genes. q-values represent the p-values corrected for multiple testing using the false discovery rate method. The last two columns in the table represent the genes used as input for the analyses and the increased allele frequency variants located in those genes, that are in common between the three isolates.

As sensitivity analysis we restricted the CPDB over-representation analysis to those variants that showed a statistically significant increase (p < 0.05) in AF compared to TSI. Although, in this case, about two-thirds of increased allele frequency variants were retained, the identified pathways remain significant at least in one of the three villages: among the 13 over-represented pathways, all of them showed a p-value < 0.05 in the three villages, and 10 pathways in Gioi and 11 in Cardile showed also a q-value < 0.05. Results of this analysis are reported in the Supplementary Table 6.

### Rare genetic disease-causing variants

Taking into account the same variants used for the analysis described in the previous section, and following a search for them in the ClinVar archive of NCBI (https://www.ncbi.nlm.nih.gov/clinvar)^23^, we found 20 variants increased in AF described as pathogenic for rare genetic diseases in at least one village: 9 variants were identified in Campora, 6 in Gioi, and 12 in Cardile. Also, 10 out of the 20 variants were monomorphic in TSI but they showed a MAF ≥ 0.0223 in at least one isolate. The list of the identified variants, together with the rare genetic pathologies for which they are responsible is reported in Table 2. For each variant, a description of its characteristics as well as those of the related disease is also reported in Supplementary Data. Although the overall reported diseases affect a large number of organs/systems, different conditions affecting the metabolism, the neurological system, and the eye emerge.

**Table 2:** Rare disease causing variants, reported as Pathogenic in ClinVar database, increased in allele frequency in at least one Cilento isolate.

Disease	Orphanet Number	Orphanet classification	Gene	Variant	Allele Frequency				Fold increase		
					Campora	Gioi	Cardile	TSI	Campora	Gioi	Cardile
2-methylbutyryl-CoA dehydrogenase deficiency	79157	inborn error of metabolism; neurological disease	ACADSB	rs58639322	**0.070**	**0.032**	/	0.005	**15.0**	**6.8**	/
Autosomal recessive isolated neurosensory deafness type DFNB	90636	otorhinolaryngologic disease	MYO15A	rs121908970	/	**0.043**	0.009	/	/	/	/
Behcet’s syndrome	117	neurological disease; skin disease; renal disease; eye disease; systemic and rheumatological disease; circulatory system disease	ADA2	rs146597836	**0.027**	/	**0.060**	0.005	**5.8**	/	**12.9**
Butyrylcholinesterase	132	inborn error of metabolism; neurological disease	BCHE	rs28933390	**0.124**	0.005	**0.043**	0.019	**6.6**	0.3	2.3
Carnitine palmitoyltransferase II deficiency	157	inborn error of metabolism; neurological disease	CPT2	rs74315294	0.005	/	**0.052**	0.005	1.2	/	**11.1**
Cerebral autosomal dominant arteriopathy with subcortical infarcts and leukoencephalopathy (CADASIL)	136	neurological disease; eye disease	NOTCH3	rs201680145	/	/	**0.129**	/	/	/	/
Corneal dystrophy Fuchs endothelial	98974	eye diseases	ZEB1	rs118020901	**0.038**	/	/	/	/	/	/
Cowden syndrome	201	gastroenterological disease; skin disease; neoplastic disease; developmental anomalies during embryogenesis	SEC. 23B	rs36023150	/	**0.048**	0.009	0.009	/	**5.1**	0.9
delta- beta Thalassemia	231237	hematological disease	HBD	rs35152987	**0.027**	/	0.009	/	/	/	/
Emery-Dreifuss muscular dystrophy	261	cardiac disease; neurological disease	SYNE1	rs119103248	0.011	/	**0.026**	0.005	2.3	/	**5.5**
Hereditary chronic pancreatitis	676	gastroenterological disease; endocrine disease	CFTR	rs1800111	**0.048**	0.011	**0.052**	/	/	/	/
Keratoconus	156071	eye disease	ZNF469	rs281865162	/	**0.085**	0.009	0.005	/	**18.2**	1.8
Leber congenital amaurosis 4	65	eye disease; ciliopathy	AIPL1	rs62637014	0.011	/	**0.043**	/	/	/	/
Leber congenital amaurosis 6	65	eye disease; ciliopathy	RPGRIP1	rs17103671	/	0.011	**0.026**	0.005	/	2.3	**5.5**
Microphthalmia syndromic 9	2470	eye disease; respiratory disease; surgical thoracic and abdominal disease; developmental anomalies during embryogenesis	STRA6	rs118203962	**0.032**	/	/	0.005	**6.9**	/	/
Odontoonychodermal dysplasia	99798	odontological disease	WNT10A	rs121908120	0.011	**0.037**	**0.043**	0.005	2.3	**8.0**	**9.2**
Primary ciliary dyskinesia	244	respiratory disease; infertility disorder; ciliopathy	RSPH1	rs138320978	0.005	**0.027**	0.017	/	/	/	/
Pseudoxanthoma elasticum	758	eye disease; skin disease; renal disease; neurological disease; cardiac disease; circulatory system disease; developmental anomalies during embryogenesis	ABCC6	rs72653706	**0.054**	0.016	**0.026**	/	/	/	/
Rare hereditary thrombophilia	217454	hematological disease; systemic and rheumatological disease; bone disease	F5	rs6025	**0.054**	/	**0.052**	0.005	**11.5**	/	**11.1**
Tyrosinemia type I	882	inborn errors of metabolism; neurological disease; hepatic disease; renal disease; neoplastic disease	FAH	rs11555096	0.016	0.005	**0.060**	0.005	3.5	1.1	**12.9**

Fold increases ≥5 and allele frequencies ≥0.0223 are reported in bold.

To confirm the increase in allele frequency of the identified variants, we directly genotyped two of them (rs72653706 and rs201680145). In particular, the variant rs72653706, selected as a representative of increased frequency in all three villages, was directly genotyped in all available samples in Cilento and showed the following allele frequencies: Campora AF = 0.027 (40 carriers on 750 genotyped individuals), Gioi AF = 0.008 (12 carriers on 720), and Cardile AF = 0.046 (50 carriers on 544). No individual was homozygous for the deleterious allele in the Cilento villages. One individual, not included in the WES study, was found to be heterozygous for the rs72653706 variant and was affected by Pseudoxanthoma Elasticum, as reported in the clinical data collected. The hypothesis that this individual is a compound heterozygous should be further verified. The variantrs201680145, responsible for CADASIL disease, and chosen due to its presence in the Cardile population, was directly genotyped on the overall Cardile sample, confirming the presence of the disease allele in this population with an AF = 0.073 (76 heterozygous and 3 homozygous individuals on 564 genotyped individuals).

### Population Genetics analyses

**Figure 4:**
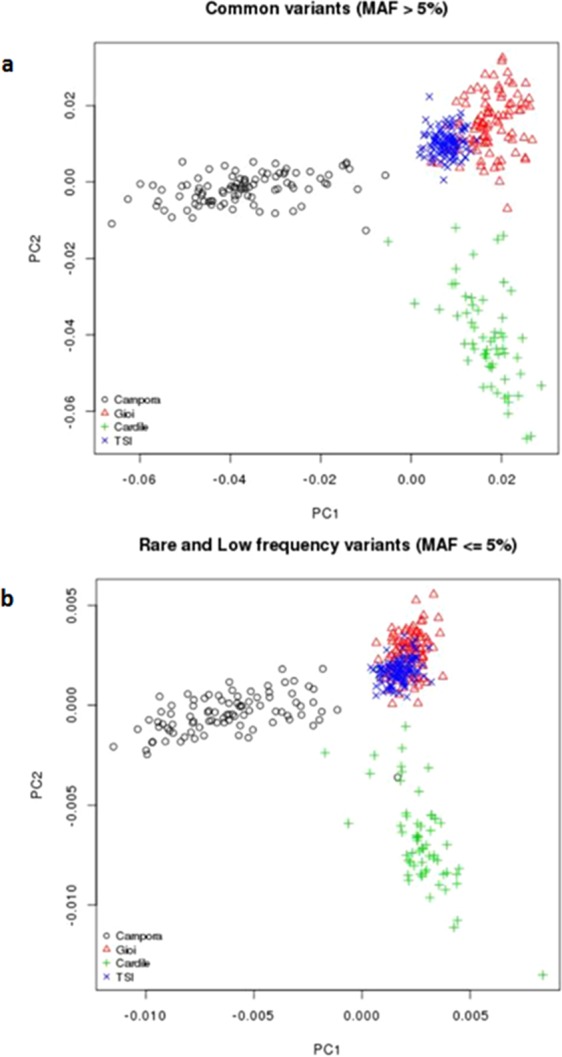
PCA analysis. Principal components analysis of Campora, Gioi and Cardile, combined with the Tuscan (TSI) population from the 1000 Genomes Phase 3 v5 reference panel. The analysis was performed using (**a**) common (MAF > 5%) and (**b**) rare and low frequency (MAF ≤ 5%) variants in common between the Cilento isolates and TSI. We compare the first and second principal components (PC1 and PC2, respectively).

To characterize the genetic differentiation of the Cilento isolates, a Principal Component Analysis (PCA) was performed including data of each village and TSI population. The analysis, both using common (MAF > 5%) and rare and low-frequency (MAF ≤ 5%) variants revealed three defined clusters corresponding to the three isolates, with the first principal component discriminating Campora and Cardile from Gioi and the second principal component separating Campora and Cardile from TSI. We observed that Gioi partially overlaps with TSI, in particular when considering rare and low frequency variants (Fig. 4a,b). Effective population size (Ne) history based on LD estimations reveals that the three isolates have substantially smaller Ne compared to the Italian general population (TSI). The analysis showed that 30,000 years ago there was a comparable size among the three isolates (about 3,500 for isolates vs 4,500 for TSI). After, while the Ne of Campora and Cardile approximately remains unchanged over time, the Ne of Gioi tends to increase but always remaining smaller than TSI. At 5,000 years ago; the isolate population sizes diverge considerably with Ne~3,000 in Campora and Cardile, Ne~5,000 in Gioi *vs* Ne~10,000 in TSI (Supplementary Fig. 1).

**Figure 5:**
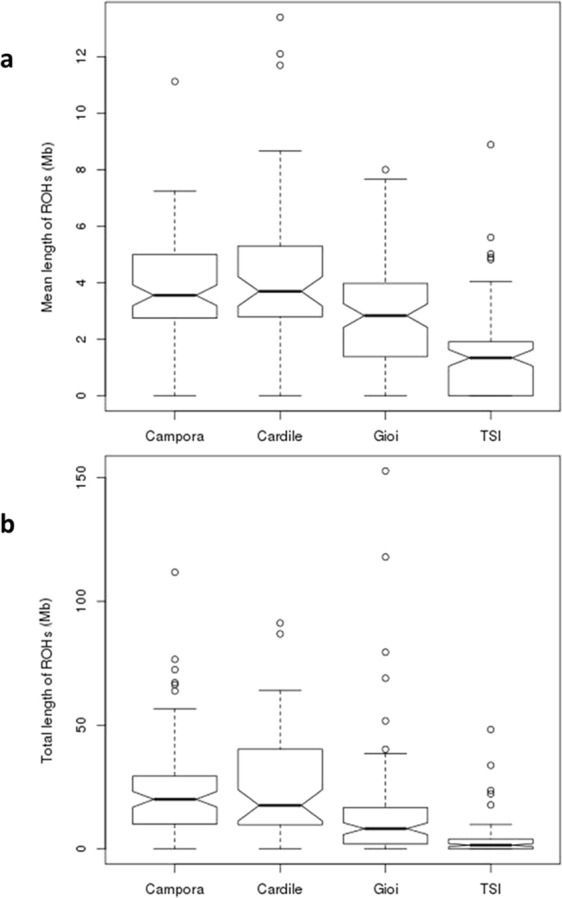
Runs Of Homozygosity (ROH) analysis. (**a**) Mean and (**b**) total length (Mb) of ROH in Campora, Gioi, Cardile and TSI populations. Only ROH with length >1 Mb are shown.

To detect autozygosity, we searched for Runs of Homozygosity (ROH). These are contiguous regions of the genome where an individual is homozygous across all sites. This analysis revealed that Campora, Cardile and Gioi show a significantly higher number of homozygous segments (5.58, 5.57 and 3.67 on average, respectively), compared to TSI (1.48, Campora-TSI p = 5.42E-17, Cardile-TSI p = 1.76E-11, Gioi-TSI p = 1.16E-5). These segments have a greater mean length in Cilento isolates compared to TSI (Campora = 3.87 Mb, Cardile = 4.34 Mb, Gioi = 2.82 Mb, TSI = 1.42 Mb; Campora-TSI p = 7.49E-20, Cardile-TSI p = 2.23E-11, Gioi-TSI p = 5.11E-8,) (Fig. 5a). Also, the total length of ROH is significantly higher in Campora (23.84 Mb), Cardile (25.38 Mb) and Gioi (14.48 Mb) compared to TSI (3.54 Mb, Campora-TSI p = 1.19E-15, Cardile-TSI p = 1.67E-10, Gioi-TSI p = 2.15E-5) (Fig. 5b).

The analysis also revealed that, while in TSI the majority of ROH show a length between 1 and 2 Mb (~60%), in Cilento populations these short segments represent less than 30% of the total, while a higher prevalence of long segments (>2 Mb) can be observed in these populations compared to TSI. This is especially evident for segments with a length >10 Mb, which occur with a frequency of 1.27% in TSI, while they reach a frequency of 7.51% in Campora, 8.98% in Cardile and 6.38% in Gioi (Supplementary Fig. 2). These results are in agreement with isolation and the presence of consanguineous unions in the history of these populations.

### Improvement of Imputation

**Figure 6:**
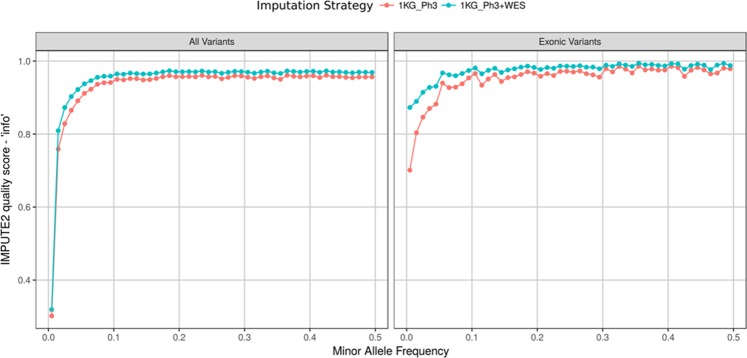
Improvement of imputation. Comparison of IMPUTE2 imputation quality score ‘info’ between two imputation strategies: firstly using the 1000 Genomes Phase3 v5 reference panel (‘1KG_Ph3’ – red) and secondly using a combination of the 1000 Genomes Phase3 v5 reference panel and a local reference panel of phased exome data from Cilento (‘1KG_Ph3 + WES’ – blue). Imputation was performed on the entirety of chromosome 10. Mean ‘info’ scores for 50 MAF bins are presented and results are split between all imputed variants (left) and only imputed exonic variants (right).

Two imputation strategies were compared on the Cilento dataset on chromosome 10: firstly using the latest release of the 1KG_Ph3 and secondly using a combination of the 1KG_Ph3 and a local reference panel of haplotypes created by phasing the Cilento WES data (we denote these two imputation strategies as ‘1KG_Ph3’ and ‘1KG_Ph3 + WES’). Over the entirety of chromosome 10, 3,921,562 and 3,923,571 variants were imputed by the strategies 1KG_Ph3 and 1KG_Ph3 + WES respectively. Indeed, including this WES local reference panel will allow the imputation of the previously discussed novel variants of Cilento. We compared imputation quality scores for variants that were neither imputed as monomorphic or present on either of the two genotyping arrays used in Cilento; our comparison involved 2,321,569 variants including 7,545 exonic variants present in the WES panel. In agreement with a recent study on simulated data in Campora^24^ and other studies examining population specific imputation panels^25–28^, our analysis revealed the addition of the WES panel improved the quality of imputation, particularly for low frequency exonic variants. Indeed, the inclusion of the WES panel led to an average increase in the ‘info’ score by 2.1% (0.96 to 0.98) across exonic variants with a MAF > 0.05 and by 15.9% (0.77 to 0.89) over exonic variants with MAF ≤ 0.05 (Fig. 6). From the set of variants used for the comparison of imputation strategies, 1KG_Ph3 + WES imputed variants had higher ‘info’ scores: the number of poorly imputed variants (‘info’ <0.4) was 1,259,194 under 1KG_Ph3 + WES compared to 1,300,113 under 1KG_Ph3 and the number of confidently imputed variants (‘info’ >0.7) was 872,425 under 1KG_Ph3 + WES compared to 756,520 under 1KG_Ph3 (+15.3% of confidently imputed variants).

## Discussion

Next-generation sequencing approaches have produced a more comprehensive survey of the genetic diversity in human population improving knowledge of population history and disease mapping studies. These studies are particularly interesting in founder populations which constitute a special resource due to the effects of bottlenecks and drift on their genetic variation. In fact, several examples of complex trait associations in isolates involve variants that are rare or at low frequency in the general population yet are shown to occur with greater frequency in the studied population isolates^29–32^. This work provides a deep description of the exonic architecture of the isolated populations of Cilento, and represents the largest exome variant analysis in a population-based study in Southern Italy. The study identifies several variants with an increased allele frequency that could be prioritized in genetic association studies and for which fewer individuals would be required to achieve sufficient power to detect association. In line with a founder effect, some mutations, responsible for rare genetic conditions, are at high frequency in Cilento isolates.

In this study we have identified 347,684 variants, the vast majority of which (over two thirds) are rare or at low frequency. This is in accordance with other previous studies on whole-exome sequencing analysis^2,4^. According to the effect of variants, each person in our study carries, on average, 319 HIGH impact variations; a lower burden of deleterious variations per person was reported in a similar study on the isolated population of VIS in Croatia^2^. However, in that case a slightly different classification of loss of function variants was used including only the stop-gained (nonsense) or splice site-disrupting (splice donor or acceptor) variants.

By comparing the Cilento sequences with data from five public databases we have identified many novel, Cilento specific variants, representing more than 11% of the total set of variations found in Cilento. This was similar to the percentage observed in an exome-sequencing study on an isolated population from the Dalmatian island of Vis (9%; where five databases were explored)^2^ and lower than the percentage reported in a whole-exome sequencing study of a French-Canadian founder population (20%; but here only the 1000 genome database was explored)^4^. As already found in other studies on isolated populations^2,10^, novel variants were enriched in rarity and functional relevance, which is expected as these variants have not fully undergone purifying selection due to the relatively recent foundation of these isolates.

The comparison of allele frequencies of the Cilento isolate variants with those from the Tuscan population, a general Italian population, highlighted the existence of variations that have drifted positively in frequency in Cilento. Interestingly, a pattern of common enriched pathways, mainly related to extracellular matrix formation and organization, was found in all the three villages. Genes related to collagen proteins and the extracellular matrix, and genes associated with the anatomy and physiology of cilium were reported to be potentially targets of positive selection in a study on mammal adaptation to environment^33^. Also, it has been reported in other two human populations that extracellular matrix genes show strong signs of balancing selection^34^. A work by Fumagalli *et al*. showed that genes coding for extracellular matrix component are implicated in an anti-viral response and can be thought of as targets of a virus-driven selective pressure^35^.

Genetic variations with increased AF in Cilento also included several rare disease causing variants responsible especially for pathologies compatible with life and reproduction for which high frequencies of carriers were observed. As these disease-variants were responsible for a number of diseases, it is unlikely that all these different conditions have been the subject of a carrier selective advantage. Therefore, although that hypothesis cannot be excluded, the results suggest that a stochastic effect of drift is most likely to represent the main force generating high carrier frequencies in Cilento. Recent studies on exome data have been focused on a survey of disease-allele carrier frequency at population level in both isolate communities and general populations revealing strong population specificity and variability of disease alleles^36,37^. In this context, our study provides a contribution to the frequency of rare disease causing variants in the Cilento region. Studies as such applied to larger exome/whole sequencing data from general population in Southern Italy, might contribute to create comprehensive panels of genetic screening of clinical interest.

We have added confirmation of the genetic isolation of the Cilento populations. Indeed, autozygosity, detected through ROH analysis on WES variants, revealed longer homozygous segments, especially in Campora and Cardile, compared to the general population, in accordance with similar findings obtained in two studies on Greek and other European isolated populations, in which ROH were called from SNP array data and whole-genome sequencing data, respectively^11,21^. The exome analysis confirmed previous findings from SNP chip data^38^ and places Cilento among the other European and Italian isolates for which WGS data are available.^11^. Furthermore, the results obtained from the PCA and the Effective Population Size analyses, confirmed a genetic differentiation of the Cilento isolates from the general Italian population. This differentiation was greater for Campora and Cardile compared to Gioi, as previously reported^12,13^. Effective Population Size analysis shows that isolates from Southern Italy differentiated from Italian general population similarly to isolates from Northern Italy^11^.

Finally, in accordance with previous studies demonstrating that the addition of a local reference panel in the genome-wide imputation procedure substantially improves the imputation quality, especially for rare and low frequency variants^24–28^, we reported a greater accuracy in the imputation especially for rare variants in Cilento when using a local reference panel. This will open up the opportunity for future GWAS for complex traits in Cilento including well imputed low frequency variants and indeed novel Cilento variants. This may potentially provide, in turn, a more complete description of the genetic architecture for such traits.

## Methods

### Participant recruitment and sample collection

We conducted exome-wide sequencing of 247 individuals from the isolated villages (Campora (N = 93), Gioi (N = 94) and Cardile (N = 60)) in the Cilento area, South Italy. The sample for this study was based on the initial cohort of 2,137 participants, which were recruited in the Genetic Park of Cilento and Vallo di Diano Project between 2003 and 2008. Sequenced samples were selected on the basis of three criteria: i) they (and both their parents) were born in one of the three Cilento villages; ii) they had been already genotyped with Illumina 370 K or OmniExpress arrays; iii) they maximize the whole sample haplotype representation, as they were selected using the procedure proposed by Uricchio^39^. We adapted Uricchio’s procedure from pedigree to exome data as follow: for the 1,617 individuals genotyped with one of the two Illumina arrays, the Identical by Descent (IBD) segments were reconstructed with GERMLINE using the 192,092 SNPs in common between the two chips. Then the segments located on exome regions were selected to calculate the exome kinship using the estimated Jacquard identity coefficients^40^. Finally, the person with the highest average kinship to the other individuals in the study was chosen and subsequently, in an iterative manner, the individuals who had the highest average kinship to the remaining unselected individuals, but who had kinship not exceeding 0.1 with any other individual already selected for sequencing, were selected.

Mean genomic kinship of the 247 individual sample was 0.007, as estimated by GenABEL library^41^.

The study design was approved by the ethics committee of Azienda Sanitaria Locale Napoli 1 and the ethics committee “Carlo Romano” University of Naples “Federico II”. The study was conducted according to the criteria set by the declaration of Helsinki and each subject signed an informed consent form before participating in the study.

### Exome capture and sequencing

Sequencing was performed at the Wellcome Trust Sanger Institute, Hinxton, Cambridge, UK. Genomic DNA (approximately 1 μg) was fragmented to an average size of 150 bp and subjected to DNA library creation using established Illumina paired-end protocols. Adapter-ligated libraries were amplified and indexed via Polymerase Chain Reaction (PCR). A portion of each library was used to create an equimolar pool comprising eight indexed libraries. Each pool was hybridised to SureSelect RNA baits (Human All Exon V5; Agilent Technologies) and sequence targets were captured and amplified in accordance with the manufacturer’s recommendations. Enriched libraries were subjected to 75 base paired-end sequencing (HiSeq 2000; Illumina) following the manufacturer’s instructions.

### Variant calling, filtering and annotation

Sequence reads were aligned with the Burrows-Wheeler Aligner (BWA, v0.5.10) algorithm on to the GRCh37 human assembly. The paired-read alignments were sorted and stored in BAM format using samtools (v0.1.18). Duplicates were marked with Picard (v1.72), and local re-alignment and Base Quality Score Recalibration were carried out using Genome Analysis Toolkit (GATK, v1.5–9, https://software.broadinstitute.org/gatk/)^42^.

After data pre-processing, a multi-sample variant-calling was performed using Samtools mpileup (v0.1.19). GATK Unified Genotyper (v1.5-32-g2761da9) was used to recall the sites discovered by samtools and to perform the Variant Quality Score Recalibration (VQSR). Single Nucleotide Variants (SNVs) and indels were filtered by minimum VQSLOD score corresponding to the truth sensitivity threshold of 99.5% and 95% respectively. Genotypes with a depth (DP) < 4 and/or a genotype quality (GQ) < 60 for that particular call, were set as null genotypes.

Called variants were annotated with dbSNP137 rsIDs and 1000 Genomes super population allele frequencies that were extracted from the final 1000 Genomes Phase 1 integrated (v3) callset.

Functional annotations were called with the Ensembl Variant Effect Predictor (http://www.ensembl.org/info/docs/tools/vep/index.html)^43^ against Ensembl 75, which provided genes and transcripts affected by the variants, location, coding consequence predictions and SIFT, PolyPhen and Condel annotations as well as GERP conservation scores. The most deleterious effect, according to severity estimated by Ensembl (https://www.ensembl.org/info/genome/variation/prediction/predicted_data.html), was assigned to each variant.

### Performance of whole-exome sequencing

Performance of whole-exome sequencing was evaluated using GATK^42^. We collected high-coverage exome sequencing data for 247 samples with a mean of 83.3 million reads per subject. When PCR duplicates were removed, 69.8 million reads (83.9%) per individual were retained.

The mean value of aligned-read depth on the target regions (~50 Mb) was ~75x (range 57–103x) with more than 85% of target regions covered by at least 30x, and 66% covered by at least 50x. Considering also the calling regions outside the targets (for a total of ~90 Mb), the mean value of depth per individual was 50 (range 39–72), with 75% of positions covered by at least at 30x.

### Per-sample and per-variant Quality control

The sample-level Quality Control (QC) criteria were: (a) genotype concordance for the overlapping set of individuals/SNPs between the Cilento genome-wide data (genotyped using Illumina 370 K or OmniExpress arrays) and whole-exome sequences. Two individuals (from Cardile population) with more than 50 discordant sites were excluded. A very high genotype concordance rate (mean 99.8%, SD 0.05%) was observed for the remaining individuals; (b) a sample call rate exclusion threshold of 90% was applied with no sample exclusion; (c) the heterozygosity exclusion threshold of ±3 standard deviations (SD) from the mean was used again with no sample exclusion; (d) outliers were searched for using multidimensional scaling analysis (MDS), plots were visually inspected and no subjects were excluded. Per-sample QC steps were performed using PLINK 1.9^44^. Samples failing sample-level QC were removed prior to performing variant-level QC.

The variant-level QC step was carried out under the following criteria: (a) only variants that were autosomal, biallelic, and polymorphic in the Cilento sample (MAC ≥ 1) were included; (b) variant call rate threshold of 95% was applied; (c) deviation from Hardy-Weinberg equilibrium (HWE) was calculated and variants falling below the threshold for HWE, exact p-value < 1 × 10^−4^, were excluded. All variants failing QC steps were removed. All subsequent analyses were performed using clean post-QCed datasets. Per-variant QC steps were performed using VCFtools (v0.1.12b).

The post-QC Cilento exome-sequence dataset contained 245 (93 from Campora, 94 from Gioi and 58 from Cardile) individuals (45.7% males) and 347,684 variants.

### Ti/Tv ratio

To assess the probability of false positives we evaluated aggregate transition-transversion (Ti/Tv) ratio for all variants that passed QC. Average Ti/Tv ratio for targeted regions over all subjects and all locations was 2.57, in accordance with the value expected in such studies.

### Cilento variant analysis

As the sequenced individuals were selected to be not closely related to each other, their relationship should not influence the allele frequency estimation. For this reason, the MAF of the variants identified in the Cilento sample was calculated by naive counting. According to their MAF, we classified the 347,684 variants in 6 categories: singletons (MAC = 1), doubletons (MAC = 2), ≤1%, 1–2%, 2–5%, >5%. The accuracy of singletons was verified by looking at allele transmission in a subset of 6 out of the 245 individuals who were part of parent-child couples for which additional whole-genome sequences were also available. On average, we observed a transmission rate of 50.5% for these variants, as expected. This result indicates that the identified singletons are unlikely to be false positive variants.

Also, sequence variants were classified into four functional impact categories, in order of decreasing severity: HIGH (Loss of Function), MODERATE, LOW, and MODIFIER, following functional annotation groups as reported by Ensembl (https://www.ensembl.org/info/genome/variation/prediction/predicted_data.html). In order to identify novel variants not reported in the reference datasets, we compared the Cilento exome data with five databases: HapMap3 release 2 (http://www.hapmap.org), 1KG_Ph3 (http://www.internationalgenome.org/), Haplotype Reference Consortium (HRC) release 1.1 (http://www.haplotype-reference-consortium.org/), dbSNP142 (https://www.ncbi.nlm.nih.gov/projects/SNP/), and ExAC release 0.3.1 (http://exac.broadinstitute.org/). To check if a Cilento variant was shared with a reference database we matched chromosome, position and both the alleles (including multiallelic variants in reference database).

### Functional enrichment of novel variants

To test for functional enrichment of Cilento novel variants, we divided all variants in two groups: those found in at least one of the five reference database (SHARED) and those private to Cilento (NOVEL). Variants were then grouped by MAF and impact categories, as described above. Two sided tests based on asymptotic normal distributions were used to compare the proportion between the SHARED and NOVEL datasets for each MAF and Impact category. A Fisher exact test was used instead for the categories containing variant counts <5. Fold enrichment was calculated with the following equation:

$${{\boldsymbol{E}}}^{M,i}=\frac{{{\boldsymbol{V}}}_{N}^{M,i}/{{\boldsymbol{t}}}_{N}^{M}}{{{\boldsymbol{V}}}_{S}^{M,i}/{{\boldsymbol{t}}}_{S}^{M}}$$

where ${{\boldsymbol{E}}}^{M,i}\,$is the fold enrichment of impact $\,i$ in $M$ MAF category; ${{\boldsymbol{V}}}_{N}^{M,i}$ and ${{\boldsymbol{V}}}_{S}^{M,i}$ are the number of variants in $M$ MAF category with impact $i$ in the NOVEL and the SHARED dataset respectively; ${{\boldsymbol{t}}}_{N}^{M}$ and ${{\boldsymbol{t}}}_{S}^{M}$ are the total number of variants in the $M$ MAF category in the NOVEL and the SHARED dataset respectively. Bonferroni corrections of the p-values were applied to account for multiple testing.

### Allele frequency comparison

To examine the genetic drift in the Cilento isolates, we compared the allele frequencies of all variants in Campora, Gioi, and Cardile (N = 93, 94, and 58 samples, respectively) against their respective frequencies in the TSI sample (N = 112). To do this, in each population, variants shared with TSI were selected to be: autosomal, biallelic (removing variants with discordant alleles), with HWE p ≥ 10^−4^ and callrate >0.95. Applying these filters 204,674, 218,270, and 187,259 variants were analysed in Campora, Gioi, and Cardile, respectively. For each variant (except for those monomorphic in TSI) we calculated the fold increase allele frequency (AF) as AF_isolate_/AF_TSI_, considering as reference allele the minor allele in TSI.

### Gene-enrichment analysis

An enrichment analysis for genes mapped by variants increased in allele frequency in each Cilento population versus the TSI population was performed using the Over-representation analysis option in ConsensusPathDB program (http://cpdb.molgen.mpg.de/). In particular, in each isolate we selected the genes mapped by all the variants that: a) showed an AF fold-increase ≥5 compared to TSI or b) were monomorphic in TSI (for which it was not possible to calculate the fold-increase value) and have a MAF ≥ 0.0223 in the isolate (this value corresponds to a fold-increase of 5 for MAC = 1 in TSI).

Pathways were considered as significantly enriched if they had q-value < 0.05 in CPDB analysis.

A sensitivity analysis for the over-represented pathways found in this analysis was also performed. In this case, among the variants previously considered we selected those showing a statistically significant enrichment in AF compared to TSI. The statistical significance of increases was calculated on the allele counts in TSI and each Cilento village, using a Fisher exact test. Only variants with a p-value < 0.05 were considered to select the genes for the sensitivity analysis.

### Rare disease-causing variants

Variants increased in allele frequency in the isolates were searched for in ClinVar (Human Variation of Clinical Significance) database (ftp://ftp.ncbi.nih.gov/pub/clinvar/vcf_GRCh37/clinvar_20170530.vcf.gz) to identify those responsible for rare genetic diseases. Chromosome, position and alleles were used to match the variants increased in allele frequency in Cilento with those included in the ClinVar. Only those variants indicated as Pathogenic in ClinVar and reported in the OrphaNet database (www.orpha.net) because responsible for rare conditions, were selected. Direct genotyping of the rs72653706 variant on the overall population sample (Campora N = 750, Gioi N = 720, Cardile N = 544) and of the rs201680145 variant on the Cardile population sample (N = 564) was performed by TaqMan assay (Bio-Rad, USA). Genotype concordance between WES and genotyping data was verified, and no inconsistencies were found.

### Population Genetics Analyses

166,496 variants in common between Cilento villages and TSI, were selected for the analyses.

A Principal Component Analysis (PCA) was performed on Campora, Gioi, Cardile, and TSI with PLINK 1.9 software^44^, using the 106,828 common (MAF > 5%) and the 59,668 rare and low-frequency (MAF ≤ 5%) variants separately.

LD-based demographic inference for the Cilento and TSI populations was performed in the NeON R-package^45^, reconstructing the long term effective population size (Ne) from 5000 to 30000 years ago.

To detect the ROH, 60,837 among the 106,828 common variants were selected to be independent using the LD pruning option of PLINK 1.9: In windows of 50 SNPs, LD is calculated between each SNP pair, if for a pair of SNPs, the LD r^2^ exceeds 0.8 then one SNP is removed. This process is then repeated by sliding the window down the chromosome in increments of five SNPs. The ROH were reconstructed in Campora, Gioi, Cardile, and TSI applying the parameters optimized for WES analysis. That is, ROH was required to comprise a minimum of 50 consecutive homozygous SNPs without any heterozygous sites (options in Plink were as follows: –homozyg-snp 50, –homozyg-window-het 0)^46^. Only ROH with a length >1 Mb were selected. For each individual included in the analysis, the total number of segments, the total length (corresponding to the sum of ROH), and the mean length (the total length divided by the total number) of ROH were calculated. T-tests were used to test the statistical significance of the differences in means between populations.

### Imputation quality

We sought to verify if the use of the Cilento WES data as a local reference panel (LRP) in addition to the 1KG_Ph3^1^ could improve the accuracy of genome-wide imputation. To do this, we performed two imputations: the first using only the 1KG_Ph3 as reference panel, the second using a combination of both the 1KG_Ph3 and a Cilento LRP of WES data (1KG_Ph3 + WES). Variants from both Illumina 370 K and OmniExpress arrays and WES on chromosome 10 were filtered for genotyping call rate ≥95% and MAF ≥ 0.01. Mendelian errors in the genotyping data were located with PLINK 1.9 and set to missing. Two individuals were excluded from this analysis for having array genotyping rates below 70%. Haplotypes were reconstructed in two groups, one for each genotyping platform. To create the LRP for Cilento, WES data and SNPs present on both genotyping arrays were merged for the 245 exome sequenced individuals with high quality genotypes^47^. Discordant genotypes were set to missing (a mean concordance of 99.6% between WES and array genotypes was observed on chromosome 10). SHAPEIT2 was used for phasing both the 370 K and OmniExpress groups, as well as the LRP. We ran SHAPEIT2 with default settings apart from the following specifications: 15 burn-in iterations, 15 pruning iterations, 35 main iterations, and the ‘duohmm’ option^48^. We also supplied the 1KG_Ph3 as a reference panel for phasing. Imputation was performed on the 370 K and OmniExpress groups using IMPUTE2^49^ with default settings, windows of size 5 Mb, and buffer regions of size 250 Kb. The ‘merge-ref-panels’ option was used to perform imputation with a combination of the 1KG_Ph3 and the phased WES LRP. For each variant imputed in both groups, the IMPUTE2 quality score (‘info’) was calculated using QCTOOL (http://www.well.ox.ac.uk/~gav/qctool_v2/) from the imputed dosages of all individuals except those in the LRP. The ‘info’ score is an “information measure” that assesses the imputation quality of a SNP. The ‘info’ takes values between 0 and 1, where higher values indicating that a SNP has been imputed with higher certainty. This serves as a proxy for a measurement of imputation accuracy^50^. A threshold of 0.4 for the info score is generally used to select well-imputed variants to be used in GWAS. We then compared ‘info’ scores from different imputation pipelines against MAFs calculated on the European populations in the 1KG_Ph3.

## Supplementary information

Supplementary Information

## Data Availability

The datasets generated during and analysed during the current study are available in the European Genome-phenome Archive (EGA) repository, https://www.ebi.ac.uk/ega/datasets/EGAD00001002195.

## References

[1] Auton A (2015). A global reference for human genetic variation. Nature.

[2] Jeroncic A (2016). Whole-exome sequencing in an isolated population from the Dalmatian island of Vis. Eur J Hum Genet.

[3] Lim ET (2014). Distribution and medical impact of loss-of-function variants in the Finnish founder population. PLoS genetics.

[4] Casals F (2013). Whole-exome sequencing reveals a rapid change in the frequency of rare functional variants in a founding population of humans. PLoS genetics.

[5] Tang D (2016). Reference genotype and exome data from an Australian Aboriginal population for health-based research. Sci Data.

[6] Belkadi A (2016). Whole-exome sequencing to analyze population structure, parental inbreeding, and familial linkage. Proc Natl Acad Sci USA.

[7] Gudbjartsson DF (2015). Large-scale whole-genome sequencing of the Icelandic population. Nat Genet.

[8] Low-Kam C (2016). Whole-genome sequencing in French Canadians from Quebec. Hum Genet.

[9] Genome of the Netherlands, C. Whole-genome sequence variation, population structure and demographic history of the Dutch population. *Nat Genet* **46**, 818–825, 10.1038/ng.3021 (2014). 10.1038/ng.3021 24974849

[10] Southam L (2017). Whole genome sequencing and imputation in isolated populations identify genetic associations with medically-relevant complex traits. Nature communications.

[11] Xue Y (2017). Enrichment of low-frequency functional variants revealed by whole-genome sequencing of multiple isolated European populations. Nature communications.

[12] Colonna V (2007). Campora: a young genetic isolate in South Italy. Hum Hered.

[13] Colonna V (2009). Comparing population structure as inferred from genealogical versus genetic information. Eur J Hum Genet.

[14] Ruggiero D (2015). Genetic variants modulating CRIPTO serum levels identified by genome-wide association study in Cilento isolates. PLoS genetics.

[15] Choi SH (2016). Six Novel Loci Associated with Circulating VEGF Levels Identified by a Meta-analysis of Genome-Wide Association Studies. PLoS genetics.

[16] van der Harst P (2012). Seventy-five genetic loci influencing the human red blood cell. Nature.

[17] Kottgen A (2013). Genome-wide association analyses identify 18 new loci associated with serum urate concentrations. Nat Genet.

[18] Barban N (2016). Genome-wide analysis identifies 12 loci influencing human reproductive behavior. Nat Genet.

[19] Kato N (2015). Trans-ancestry genome-wide association study identifies 12 genetic loci influencing blood pressure and implicates a role for DNA methylation. Nat Genet.

[20] Gieger C (2011). New gene functions in megakaryopoiesis and platelet formation. Nature.

[21] Panoutsopoulou K (2014). Genetic characterization of Greek population isolates reveals strong genetic drift at missense and trait-associated variants. Nature communications.

[22] Kamburov A, Wierling C, Lehrach H, Herwig R (2009). ConsensusPathDB–a database for integrating human functional interaction networks. Nucleic Acids Res.

[23] Landrum MJ (2014). ClinVar: public archive of relationships among sequence variation and human phenotype. Nucleic Acids Res.

[24] Herzig AF (2018). Strategies for phasing and imputation in a population isolate. Genet Epidemiol.

[25] Deelen P (2014). Improved imputation quality of low-frequency and rare variants in European samples using the ‘Genome of The Netherlands’. Eur J Hum Genet.

[26] Surakka I (2010). Founder population-specific HapMap panel increases power in GWA studies through improved imputation accuracy and CNV tagging. Genome Res.

[27] Pistis G (2015). Rare variant genotype imputation with thousands of study-specific whole-genome sequences: implications for cost-effective study designs. Eur J Hum Genet.

[28] Mitt M (2017). Improved imputation accuracy of rare and low-frequency variants using population-specific high-coverage WGS-based imputation reference panel. Eur J Hum Genet.

[29] Holm H (2011). A rare variant in MYH6 is associated with high risk of sick sinus syndrome. Nat Genet.

[30] Tachmazidou I (2013). A rare functional cardioprotective APOC3 variant has risen in frequency in distinct population isolates. Nature communications.

[31] Lencz T (2013). Genome-wide association study implicates NDST3 in schizophrenia and bipolar disorder. Nature communications.

[32] Steri M (2017). Overexpression of the Cytokine BAFF and Autoimmunity Risk. The New England journal of medicine.

[33] Yudin NS, Larkin DM, Ignatieva EV (2017). A compendium and functional characterization of mammalian genes involved in adaptation to Arctic or Antarctic environments. BMC genetics.

[34] Andres AM (2009). Targets of balancing selection in the human genome. Molecular biology and evolution.

[35] Fumagalli M (2010). Genome-wide identification of susceptibility alleles for viral infections through a population genetics approach. PLoS genetics.

[36] Chong JX, Ouwenga R, Anderson RL, Waggoner DJ, Ober C (2012). A population-based study of autosomal-recessive disease-causing mutations in a founder population. Am J Hum Genet.

[37] Fujikura K (2016). Global Carrier Rates of Rare Inherited Disorders Using Population Exome Sequences. PLoS One.

[38] Joshi PK (2015). Directional dominance on stature and cognition in diverse human populations. Nature.

[39] Uricchio LH, Chong JX, Ross KD, Ober C, Nicolae DL (2012). Accurate imputation of rare and common variants in a founder population from a small number of sequenced individuals. Genet Epidemiol.

[40] Jacquard A (1966). Logique du calcul des coefficients d’identité entre deux individus. Population (French Edition).

[41] Aulchenko YS, Ripke S, Isaacs A, van Duijn CM (2007). GenABEL: an R library for genome-wide association analysis. Bioinformatics.

[42] McKenna A (2010). The Genome Analysis Toolkit: a MapReduce framework for analyzing next-generation DNA sequencing data. Genome Res.

[43] McLaren W (2016). The Ensembl Variant Effect Predictor. Genome Biol.

[44] Purcell S (2007). PLINK: a tool set for whole-genome association and population-based linkage analyses. Am J Hum Genet.

[45] Mezzavilla MG, Neon S (2015). An R package to estimate human effective population size and divergence time from patterns of linkage disequilibrium between SNPs. J Comput Sci Syst Biol.

[46] Magi A (2014). H3M2: detection of runs of homozygosity from whole-exome sequencing data. Bioinformatics.

[47] Joshi PK (2013). Local exome sequences facilitate imputation of less common variants and increase power of genome wide association studies. PLoS One.

[48] O’Connell J (2014). A general approach for haplotype phasing across the full spectrum of relatedness. PLoS genetics.

[49] Howie BN, Donnelly P, Marchini J (2009). A flexible and accurate genotype imputation method for the next generation of genome-wide association studies. PLoS genetics.

[50] Marchini J, Howie B (2010). Genotype imputation for genome-wide association studies. Nature reviews. Genetics.

