# Supplementary material for: Whole-Exome Sequencing in the Isolated Populations of Cilento from South Italy
Source: Sci Rep. 2019 Mar 11;9:4059. doi: 10.1038/s41598-019-41022-6 (PMC6411969; doi:10.1038/s41598-019-41022-6)
Supplement: Supplementary file 1 — Supplementary Information [file 41598_2019_41022_MOESM1_ESM.docx]

**Supplementary Data**

**for**

## Whole-Exome Sequencing in the Isolated Populations of Cilento from South Italy

T. Nutile^1^*, D. Ruggiero^1,2^*, A.F. Herzig^3,4^, A. Tirozzi^2^, S. Nappo^5^, R. Sorice^1^, F. Marangio^1^, C. Bellenguez^6,7,8^, A-L Leutenegger^3,4^, and M. Ciullo^1,2^

*^1^Institute of Genetics and Biophysics A. Buzzati-Traverso - CNR, Naples, Italy;*

*^2^IRCCS Neuromed, Pozzilli, Isernia, Italy;*

*^3^Inserm, UMR 946, Genetic variation and Human diseases, F-75010 Paris, France;*

*^4^Université Paris-Diderot, Sorbonne Paris Cité, UMR946, F-75010 Paris, France;*

*^5^AORN Santobono - Pausilipon Hospital, Naples, Italy*

*^6^Inserm, U1167, RID-AGE - Risk factors and molecular determinants of aging-related diseases, F-59000 Lille, France;*

*^7^Institut Pasteur de Lille , F-59000 Lille, France;*

*^8^Univ. Lille, U1167 - Excellence Laboratory LabEx DISTALZ, F-59000 Lille, France;*

**Description of the rare genetic disease-causing variants showing an increase in allele frequency in Cilento**

The variant rs72653706 is a stop-gained variant located in the *ABCC6* gene, which is responsible for the pseudoxanthoma elasticum disease, a multisystem disorder characterized by aberrant mineralization of soft connective tissue resulting in fragmentation of elastic fibers, involving primarily in the skin, eyes and cardiovascular gastrointestinal systems[^1^](#_ENREF_1). The disease has an autosomal recessive inheritance. The variant rs72653706, which causes the pathology both in homozygosity and in compound heterozygosity, is the most common mutation in affected individuals of European ancestry[^2^](#_ENREF_2). The deleterious allele of the rs72653706 variant is not present in the TSI samples, while its frequency reaches 0.054 (Minor Allele Count, MAC=10) in Campora and to 0.026 (MAC=3) in Cardile, with a lower frequency in Gioi (0.016, MAC=3). Five variants identified in the Cilento villages are considered causative for diseases principally affecting eyes. The variant rs118020901, located in an evolutionarily conserved residue of the ZEB1 gene, has only been found in Campora population, with a frequency of 3.8% (MAC=7), while it is not present in Gioi, Cardile, and TSI. This variant is responsible for Fuchs endothelial corneal dystrophy (FECD), the most common genetic disorder of the corneal endothelium[^3^](#_ENREF_3). Also, the variant rs281865162, an in frame deletion in the ZNF269 gene associated with Keratoconus[^4^](#_ENREF_4), has been found as a singleton in Cardile and TSI and is not present in Campora, while it reaches a MAF of 0.085 in Gioi (MAC=16). The variant rs118203962, a missense variant in the STRA6 gene, is responsible for Microphthalmia syndromic 9, a rare ocular disease, characterized by very small eyes or not clinically visible at all and is associated with other malformations of the heart, kidneys and lungs[^5^](#_ENREF_5). This variant, has a MAF of 0.005 in TSI and increases up to 0.03 in Campora (MAC=6), but is not present in Gioi and Cardile. Finally, the variants rs62637014 and rs17103671, located in the AIPL1 and the RPGRIP1 genes respectively have been both associated with Leber congenital amaurosis, a retinal dystrophy causing blindness[^6^](#_ENREF_6)^,^[^7^](#_ENREF_7), also classified as a ciliopathy. These variants have been found increased in frequency in Cardile (MAF=0.043 and 0.026, MAC=5 and 3), while are rare or not present in the other isolates and the Italian general population.

Another variant causing a ciliopathy is the rs138320978, a stop-gained mutation in the RSPH1 gene. Mutations in this gene result in Kartagener syndrome, a primary ciliary dyskinesia characterized by situ inversus totalis, sinopulmonary infection and subfertility. In particular the identified variant, which creates a stop codon in position 29 (E29X), leading to a severely truncated protein, has been found as responsible for the pathology both in homozygous and in compound heterozygous patients[^8^](#_ENREF_8). It shows a MAF of 0.027 in Gioi (MAC=5), 0.017 in Cardile (MAC=2), is a singleton in Campora while it is not present in TSI.

Four identified variants are reported as causative of metabolic disorders. In particular, the rs58639322, in the ACADSB gene, is a missense variant responsible for the 2-methylbutyryl-CoA dehydrogenase deficiency, an autosomal recessive disorder of impaired isoleucine degradation[^9^](#_ENREF_9). This variant has a MAF of 0.07 (MAC=13) in Campora, 0.032 (MAC=6) in Gioi, is a singleton in TSI and it is monomorphic in Cardile. The variant rs28933390 in the BCHE gene, coding for the butyrylcholinesterase, is a missense variant causing the deficiency of this enzyme, determining a metabolic disorder characterized by prolonged apnoea after the use of certain anesthetic drugs, including succinylcholine^[10](#_ENREF_10)^. The variant has a low frequency in TSI (MAF=0.019, MAC=4), and is present as singleton in Gioi, but it reaches a MAF of 0.04 (MAC=5) in Cardile and, notably, 0.12 in Campora (MAC=23). The variant rs74315294 is located in the CPT2 gene, coding for the carnitine palmitoyltransferase 2, an enzyme that participates in fatty acid oxidation. Mutations in the gene causes a deficit of the enzyme, leading to three different forms of the disorder, the lethal neonatal, the severe infantile hepatocardiomuscular, and the myopathic. In particular, the variant rs74315294 causes the latter form of the disease characterized by exercise-induced muscle pain and weakness, sometimes associated with myoglobinuria^[11](#_ENREF_11)^. This variant, having a MAF of 0.052 in Cardile (MAC=6), is not present in Gioi, and has been detected as singleton in TSI and Campora. The variant rs11555096, located in the Fumarylacetoacetate Hydrolase (FAH) gene, causes a deficit of the enzyme which is associated with an hereditary Tyrosinemia, characterized by progressive liver disease, renal tubular dysfunction and porphyria-like crises[^12^](#_ENREF_12). This variant is very rare in TSI and Gioi, and more common in Campora (MAF=0.016, MAC=3) and in Cardile (MAF=0.06, MAC=7).

Two variants are responsible for hematological diseases. The variant rs6025 is located in the coagulation factor 5 (F5) gene, essential for the blood coagulation cascade. The mutation, commonly referred to as “factor V Leiden”, causes Arg-to-Gln change which prevents inactivation of factor V by protein C, leading to the most common hereditary hypercoagulability disorder amongst Europeans[^13^](#_ENREF_13). This variant, which is a singleton in TSI, was found over 10-fold increased in frequency in Campora (MAF=0.054, MAC=10) and Cardile (MAF=0.052, MAC=6). The variant rs35152987, located in first nucleotide of codon 27 of the delta-globin gene, has been found as cause of delta-thalassemia in different families from Sardinia and South Italy[^14^](#_ENREF_14)^,^[^15^](#_ENREF_15). In our dataset, it has a frequency of 0.009 in Cardile (MAC=1) and reaches 0.027 in Campora (MAC=6), while has not been identified in TSI and in Gioi.

Two other variants are associated with gastroenterological diseases. The variant rs1800111 is a missense variant located in a highly conserved residue in transmembrane domain 9 of CFTR gene and causes hereditary pancreatitis[^16^](#_ENREF_16). This variant, which has not been found in TSI, reaches a frequency of 0.011 in Gioi (MAC=2) and of about 0.05 in Campora (MAC=9) and Cardile (MAC=6). The variant rs36023150, which is a rare in TSI (MAF=0.009), was found at the same frequency in Cardile and was not identified in Campora, while in Gioi reaches a frequency of 0.048 (MAC=2). It is a missense variant located in the SEC23B gene, and causes Cowden syndrome, a genodermatosis characterized by the presence of multiple hamartomas in various tissues and an increased risk for malignancies of the breast, thyroid, endometrium, kidney, and colorectum[^17^](#_ENREF_17).

The variant rs121908970 is a missense variant located in the gene coding for Myosin 15A which causes an autosomal recessive form of congenital deafness[^18^](#_ENREF_18). This variant has a MAF of 0.043 in Gioi (MAC=8), it is singleton in Cardile, while has not been identified in Campora and TSI.

The variant rs201680145 is not present in TSI, Campora, and Gioi populations, but its frequency is increased up to 13% in Cardile (MAC=15). It is located in the NOTCH3 gene, whose alterations cause cerebral autosomal dominant arteriopathy with subcortical infarcts and leukoencephalopathy (CADASIL)[^19^](#_ENREF_19)^,^[^20^](#_ENREF_20).

The variant rs146597836, located in the adenosine deaminase 2 (ADA2) gene, is a missense mutation found associated with Behçet disease, a chronic multisystemic vasculitis characterized by mucocutaneous lesions, as well as articular, vascular, ocular, and central nervous system manifestations[^21^](#_ENREF_21). The variant is a singleton in TSI, and it has not been identified in Gioi, while it reaches a frequency of 0.027 in Campora (MAC=5) and of 0.06 in Cardile (MAC=7).

The variant rs121908120, found as singleton in TSI, and at a frequency of 0.011 in Campora (MAC=2), 0.037 in Gioi (MAC=7), and 0.043 in Cardile (MAC=5), is a mutation located in an evolutionarily conserved residue of the WNT10A gene, a member of the WNT gene family. This variation has been found in individuals affected by odontoonychodermal dysplasia, a form of ectodermal dysplasia characterized by hyperkeratosis and hyperhidrosis of the palms and soles, atrophic malar patches, hypodontia, conical teeth, onychodysplasia, and dry and sparse hair[^22^](#_ENREF_22).

Finally, the variant rs119103248, located in the SYNE1 gene, has been identified in an individual affected by Emery-Dreifuss muscular dystrophy 4[^23^](#_ENREF_23). Although it has been found as singleton in TSI, and is not present in Gioi, it has a frequency of 0.011 in Campora (MAC=2) and of 0.026 in Cardile (MAC=3).

**SUPPLEMENTARY TABLES**

|  |  | **Functional Impact** | | | |  |
| --- | --- | --- | --- | --- | --- | --- |
|  |  | **HIGH** | **MODERATE** | **LOW** | **MODIFIER** | **Total MAF category** |
| **Minor Allele Frequency (MAF)** | **SINGLETONS** | 1,102 (0.32) | 17,437 (5.01) | 12,381 (3.56) | 40,929 (11.77) | 71,849 (20.66) |
|  | **DOUBLETONS** | 487 (0.14) | 8,422 (2.42) | 6,217 (1.79) | 23,469 (6.75) | 38,595 (11.10) |
|  | **≤1%** | 499 (0.14) | 8,861 (2.55) | 6,776 (1.95) | 25,804 (7.42) | 41,940 (12.06) |
|  | **1-2%** | 445 (0.13) | 8,495 (2.44) | 7,106 (2.04) | 27,300 (7.85) | 43,346 (12.47) |
|  | **2-5%** | 311 (0.09) | 6,938 (1.99) | 6,731 (1.93) | 24,707 (7.11) | 38,687 (11.13) |
|  | **>5%** | 718 (0.21) | 17,506 (5.03) | 21,343 (6.14) | 73,700 (21.20) | 113,267 (32.58) |
|  | **Total Impact category** | 3,562 (1.02) | 67,659 (19.46) | 60,554 (17.42) | 215,909 (62.10) | 347,684 (100) |

**Supplementary Table S1.** The count (and percentage in brackets) of variants found in the Cilento whole-exome sequencing study, categorized by functional impact and minor allele frequency.

|  | | **Shared variants** | | | | | **Novel variants** |
| --- | --- | --- | --- | --- | --- | --- | --- |
| **IMPACT** | **MAF** | **Found in 5 database** | **Found in 4 database** | **Found in 3 database** | **Found in 2 database** | **Found in 1 database** |  |
| **HIGH** | **SINGLETONS** | 6 | 205 | 151 | 89 | 264 | 387 |
|  | **DOUBLETONS** | 6 | 104 | 81 | 36 | 117 | 143 |
|  | **≤1%** | 9 | 139 | 85 | 43 | 111 | 112 |
|  | **1-2%** | 18 | 163 | 97 | 30 | 72 | 65 |
|  | **2-5%** | 24 | 170 | 75 | 16 | 19 | 7 |
|  | **>5%** | 199 | 285 | 215 | 17 | - | 2 |
| **MODERATE** | **SINGLETONS** | 348 | 6178 | 1963 | 1715 | 3386 | 3847 |
|  | **DOUBLETONS** | 214 | 3581 | 950 | 823 | 1445 | 1409 |
|  | **≤1%** | 301 | 4403 | 925 | 713 | 1317 | 1202 |
|  | **1-2%** | 581 | 5188 | 742 | 513 | 823 | 648 |
|  | **2-5%** | 1141 | 4986 | 369 | 135 | 181 | 126 |
|  | **>5%** | 8008 | 8757 | 596 | 132 | 11 | 2 |
| **LOW** | **SINGLETONS** | 384 | 5353 | 1369 | 1184 | 2090 | 2001 |
|  | **DOUBLETONS** | 228 | 3106 | 706 | 564 | 813 | 800 |
|  | **≤1%** | 287 | 3837 | 668 | 488 | 815 | 681 |
|  | **1-2%** | 527 | 4749 | 559 | 325 | 533 | 413 |
|  | **2-5%** | 1145 | 4963 | 305 | 128 | 118 | 72 |
|  | **>5%** | 9884 | 10649 | 703 | 102 | 3 | 2 |
| **MODIFIER** | **SINGLETONS** | 324 | 7642 | 12128 | 3632 | 4321 | 12882 |
|  | **DOUBLETONS** | 214 | 4987 | 8228 | 1845 | 2103 | 6092 |
|  | **≤1%** | 316 | 6231 | 10091 | 1917 | 1914 | 5335 |
|  | **1-2%** | 548 | 8109 | 12474 | 1710 | 1307 | 3152 |
|  | **2-5%** | 1299 | 9338 | 12130 | 1054 | 333 | 553 |
|  | **>5%** | 11814 | 32090 | 27309 | 2248 | 226 | 13 |
| **Total Variant count** | | 37825 | 135213 | 92919 | 19459 | 22322 | 39946 |

**Supplementary Table S2.** The count of known and novel Cilento variants in each functional impact group categorized by minor allele frequency. For known variants, the number of reference database in which they have been found is also reported.

|  |  | **NOVEL variants** | | | **SHARED variants** | | | **Enrichment** | **z** | **p-value** |
| --- | --- | --- | --- | --- | --- | --- | --- | --- | --- | --- |
| **IMPACT** | **MAF category** | **Variant count** | **Variant count in MAF category** | **Proportion** | **Variant count** | **Variant count in MAF category** | **Proportion** |  |  |  |
| **HIGH** | **SINGLETONS** | 387 | 19117 | 0.020 | 715 | 52732 | 0.014 | 1.493 | 6.443 | **2.81E-09** |
|  | **DOUBLETONS** | 143 | 8444 | 0.017 | 344 | 30151 | 0.011 | 1.484 | 4.021 | **1.39E-03** |
|  | **≤1%** | 112 | 7330 | 0.015 | 387 | 34610 | 0.011 | 1.366 | 2.939 | 0.079 |
|  | **1-2%** | 65 | 4278 | 0.015 | 380 | 39068 | 0.010 | 1.562 | 3.368 | **0.018** |
|  | **2-5%** | 7 | 758 | 0.009 | 304 | 37929 | 0.008 | 1.152 | 0.372 | 1 |
|  | **>5%** | 2 | 19 | 0.105 | 716 | 113248 | 0.006 | 16.649 | 16.647* | 0.184 |
| **MODERATE** | **SINGLETONS** | 3847 | 19117 | 0.201 | 13590 | 52732 | 0.258 | 0.781 | -28.910 | ***1.58E-53*** |
|  | **DOUBLETONS** | 1409 | 8444 | 0.167 | 7013 | 30151 | 0.233 | 0.717 | -18.762 | ***7.75E-37*** |
|  | **≤1%** | 1202 | 7330 | 0.164 | 7659 | 34610 | 0.221 | 0.741 | -17.581 | ***2.24E-26*** |
|  | **1-2%** | 648 | 4278 | 0.151 | 7847 | 39068 | 0.201 | 0.754 | -12.542 | ***2.69E-13*** |
|  | **2-5%** | 126 | 758 | 0.166 | 6812 | 37929 | 0.180 | 0.926 | -5.794 | 1 |
|  | **>5%** | 2 | 19 | 0.105 | 17504 | 113248 | 0.155 | 0.681 | 0.681* | 1 |
| **LOW** | **SINGLETONS** | 2001 | 19117 | 0.105 | 10380 | 52732 | 0.197 | 0.532 | -15.606 | ***2.17E-182*** |
|  | **DOUBLETONS** | 800 | 8444 | 0.095 | 5417 | 30151 | 0.180 | 0.527 | -12.925 | ***3.68E-77*** |
|  | **≤1%** | 681 | 7330 | 0.093 | 6095 | 34610 | 0.176 | 0.528 | -10.919 | ***8.22E-68*** |
|  | **1-2%** | 413 | 4278 | 0.097 | 6693 | 39068 | 0.171 | 0.564 | -7.725 | ***1.06E-34*** |
|  | **2-5%** | 72 | 758 | 0.095 | 6659 | 37929 | 0.176 | 0.541 | -0.950 | ***1.65E-07*** |
|  | **>5%** | 2 | 19 | 0.105 | 21341 | 113248 | 0.188 | 0.559 | 0.559* | 1 |
| **MODIFIER** | **SINGLETONS** | 12882 | 19117 | 0.674 | 28047 | 52732 | 0.532 | 1.267 | 33.964 | **1.79E-251** |
|  | **DOUBLETONS** | 6092 | 8444 | 0.721 | 17377 | 30151 | 0.576 | 1.252 | 24.145 | **2.02E-127** |
|  | **≤1%** | 5335 | 7330 | 0.728 | 20469 | 34610 | 0.591 | 1.231 | 21.806 | **4.87E-104** |
|  | **1-2%** | 3152 | 4278 | 0.737 | 24148 | 39068 | 0.618 | 1.192 | 15.264 | **3.20E-51** |
|  | **2-5%** | 553 | 758 | 0.730 | 24154 | 37929 | 0.637 | 1.146 | 5.262 | **3.42E-06** |
|  | **>5%** | 13 | 19 | 0.684 | 73687 | 113248 | 0.651 | 1.052 | 0.307 | 1 |

**Supplementary Table S3.** Distribution of functional consequences for the Cilento novel variants compared to those shared with at least one database. For each functional impact group the variants are categorized in Minor Allele Frequency classes. The variant count is the number of variants in the given impact and MAF category. The variant count in each MAF category is the total number of variants in the MAF group. The Proportion is calculated as Variant count/Variant count in MAF category. Enrichment is calculated as Proportion of Cilento novel variants/Proportion of shared variants; z is the statistics of the two-sided test based on asymptotic normal distribution; * A Fisher exact test was used for the categories containing variant counts <5; p-value is Bonferroni corrected; significant enrichments (p-value < 0.05) are reported in bold underlined, significant depletions (p-value < 0.05) are reported in bold italic.

| **a** |  |  | **IMPACT / Allele Frequency in Campora** | | | | | | | | | | | | |
| --- | --- | --- | --- | --- | --- | --- | --- | --- | --- | --- | --- | --- | --- | --- | --- |
|  | **Fold Allele Frequency Increase (Campora vs TSI)** | **Variant count** | **HIGH** | | | **MODERATE** | | | **LOW** | | | **MODIFIER** | | | |
|  |  |  | **≤1%** | **1-5%** | **>5%** | **≤1%** | **1-5%** | **>5%** | **≤1%** | **1-5%** | **>5%** | **≤1%** | **1-5%** | **>5%** | |
|  | **Monomorphic in TSI** | 25,486 | 92 | 117 | 7 | 2,149 | 2,799 | 188 | 1,887 | 2,403 | 147 | 6,481 | 8,707 | 509 | |
|  | **<5** | 88,652 | 46 | 192 | 402 | 939 | 4,052 | 9,390 | 851 | 3,849 | 11,521 | 3,072 | 14,548 | 39,790 | |
|  | **5-9** | 3,587 | - | 25 | 11 | - | 451 | 241 | - | 392 | 186 | - | 1,489 | 792 | |
|  | **10-14** | 780 | - | 2 | 4 | - | 43 | 124 | - | 45 | 93 | - | 128 | 341 | |
|  | **15-19** | 203 | - | - | - | - | - | 44 | - | - | 32 | - | - | 127 | |
|  | **20-24** | 57 | - | - | - | - | - | 13 | - | - | 8 | - | - | 36 | |
|  | **≥25** | 30 | - | - | - | - | - | 3 | - | - | 7 | - | - | 20 | |
|  | **TOT** | 118,795 | 138 | 336 | 424 | 3,088 | 7,345 | 10,003 | 2,738 | 6,689 | 11,994 | 9,553 | 24,872 | 41,615 | |
|  |  |  |  |  |  |  |  |  |  |  |  |  |  |  |  |
| **b** |  |  | **IMPACT / Allele Frequency in Gioi** | | | | | | | | | | | | |
|  | **Fold Allele Frequency Increase (Gioi vs TSI)** | **Variant count** | **HIGH** | | | **MODERATE** | | | **LOW** | | | **MODIFIER** | | | |
|  |  |  | **≤1%** | **1-5%** | **>5%** | **≤1%** | **1-5%** | **>5%** | **≤1%** | **1-5%** | **>5%** | **≤1%** | **1-5%** | **>5%** | |
|  | **Monomorphic in TSI** | 33,130 | 165 | 107 | 5 | 3,439 | 3,164 | 67 | 3,053 | 2,722 | 57 | 10,191 | 9,947 | 213 | |
|  | **<5** | 93,806 | 59 | 218 | 390 | 1,458 | 5,045 | 9,453 | 1,275 | 4,588 | 11,420 | 4,350 | 17,503 | 38,047 | |
|  | **5-9** | 3,047 | - | 25 | 6 | - | 471 | 121 | - | 422 | 112 | - | 1,486 | 404 | |
|  | **10-14** | 486 | - | - | 1 | - | 30 | 60 | - | 23 | 56 | - | 101 | 215 | |
|  | **15-19** | 83 | - | - | 1 | - | - | 17 | - | - | 18 | - | - | 47 | |
|  | **20-24** | 10 | - | - | - | - | - | 3 | - | - | 1 | - | - | 6 | |
|  | **≥25** | 7 | - | - | - | - | - | 2 | - | - | 1 | - | - | 4 | |
|  | **TOT** | 130,569 | 224 | 350 | 403 | 4,897 | 8,710 | 9,723 | 4,328 | 7,755 | 11,665 | 14,541 | 29,037 | 38,936 | |
|  |  |  |  |  |  |  |  |  |  |  |  |  |  |  |  |
| **c** |  |  | **IMPACT / Allele Frequency in Cardile** | | | | | | | | | | | | |
|  | **Fold Allele Frequency Increase (Cardile vs TSI)** | **Variant count** | **HIGH** | | | **MODERATE** | | | **LOW** | | | **MODIFIER** | | | |
|  |  |  | **≤1%** | **1-5%** | **>5%** | **≤1%** | **1-5%** | **>5%** | **≤1%** | **1-5%** | **>5%** | **≤1%** | **1-5%** | **>5%** | |
|  | **Monomorphic in TSI** | 20,203 | 112 | 67 | 13 | 2,114 | 1,637 | 248 | 1,900 | 1,415 | 250 | 6,433 | 5,211 | 803 | |
|  | **<5** | 81,338 | 57 | 138 | 379 | 1,024 | 2,822 | 9,590 | 866 | 2,642 | 11,601 | 3,134 | 9,869 | 39,216 | |
|  | **5-9** | 4,569 | - | 24 | 19 | - | 548 | 337 | - | 474 | 299 | - | 1,796 | 1,072 | |
|  | **10-14** | 1,051 | - | - | 13 | - | - | 207 | - | - | 193 | - | - | 638 | |
|  | **15-19** | 223 | - | - | 3 | - | - | 46 | - | - | 34 | - | - | 140 | |
|  | **20-24** | 93 | - | - | 1 | - | - | 20 | - | - | 13 | - | - | 59 | |
|  | **≥25** | 35 | - | - | - | - | - | 7 | - | - | 10 | - | - | 18 | |
|  | **TOT** | 107,512 | 169 | 229 | 428 | 3,138 | 5,007 | 10,455 | 2,766 | 4,531 | 12,400 | 9,567 | 16,876 | 41,946 | |

**Supplementary Table S4.** Allele frequency fold increases in Campora (a), Gioi (b) and Cardile (c) compared to the Tuscan (TSI) population. For each variant the increase is calculated considering the minor allele in TSI as the reference allele. The variants are grouped by their functional impact and by the frequency of the reference allele in each isolate.

| **a** | **Pathway name** | **Pathway size** | **Genes**  **contained** | **p-value** | **q-value** | **Pathway**  **source** |
| --- | --- | --- | --- | --- | --- | --- |
|  | ***Collagen chain trimerization*** | ***44*** | ***33 (75.0%)*** | ***4.57E-09*** | ***1.73E-05*** | ***Reactome*** |
|  | ***Collagen biosynthesis and modifying enzymes*** | ***67*** | ***42 (62.7%)*** | ***1.78E-07*** | ***1.39E-04*** | ***Reactome*** |
|  | ***Collagen formation*** | ***91*** | ***53 (58.2%)*** | ***1.56E-07*** | ***1.39E-04*** | ***Reactome*** |
|  | EGFR1 | 455 | 195 (43.0%) | 1.83E-07 | 1.39E-04 | NetPath |
|  | ***Extracellular matrix organization*** | ***293*** | ***136 (46.4%)*** | ***8.15E-08*** | ***1.39E-04*** | ***Reactome*** |
|  | ***Stimuli-sensing channels*** | ***102*** | ***56 (54.9%)*** | ***1.03E-06*** | ***6.49E-04*** | ***Reactome*** |
|  | ***Axon guidance*** | ***357*** | ***154 (43.3%)*** | ***2.53E-06*** | ***1.36E-03*** | ***Reactome*** |
|  | ***Transport of small molecules*** | ***666*** | ***265 (39.9%)*** | ***3.17E-06*** | ***1.50E-03*** | ***Reactome*** |
|  | Wnt Signaling Pathway | 51 | 32 (62.7%) | 5.03E-06 | 2.11E-03 | Wikipathways |
|  | Human papillomavirus infection - Homo sapiens | 319 | 137 (42.9%) | 1.39E-05 | 5.25E-03 | KEGG |
|  | ***Integrin*** | ***124*** | ***62 (50.0%)*** | ***1.67E-05*** | ***5.36E-03*** | ***INOH*** |
|  | ***Protein digestion and absorption - Homo sapiens*** | ***90*** | ***48 (53.3%)*** | ***1.70E-05*** | ***5.36E-03*** | ***KEGG*** |
|  | ***Focal adhesion - Homo sapiens (human)*** | ***199*** | ***91 (45.7%)*** | ***2.27E-05*** | ***6.58E-03*** | ***KEGG*** |
|  | ***ECM-receptor interaction - Homo sapiens*** | ***82*** | ***44 (53.7%)*** | ***3.10E-05*** | ***8.36E-03*** | ***KEGG*** |
|  | Developmental Biology | 618 | 240 (38.9%) | 7.08E-05 | 1.67E-02 | Reactome |
|  | PI3K-Akt Signaling Pathway | 340 | 141 (41.6%) | 7.03E-05 | 1.67E-02 | Wikipathways |
|  | ***Beta1 integrin cell surface interactions*** | ***66*** | ***36 (54.5%)*** | ***1.02E-04*** | ***2.14E-02*** | ***PID*** |
|  | Ion channel transport | 179 | 81 (45.3%) | 9.70E-05 | 2.14E-02 | Reactome |
|  | Ebola Virus Pathway on Host | 81 | 42 (51.9%) | 1.31E-04 | 2.48E-02 | Wikipathways |
|  | BCR | 129 | 61 (47.3%) | 1.54E-04 | 2.78E-02 | NetPath |
|  | PI3K-Akt signaling pathway - Homo sapiens | 352 | 143 (40.7%) | 1.98E-04 | 3.40E-02 | KEGG |
|  | TRP channels | 25 | 17 (68.0%) | 2.12E-04 | 3.49E-02 | Reactome |
|  | ***Vesicle-mediated transport*** | ***620*** | ***237 (38.2%)*** | ***2.73E-04*** | ***4.29E-02*** | ***Reactome*** |
|  |  |  |  |  |  |  |
| **b** | **Pathway name** | **Pathway size** | **Genes**  **contained** | **p-value** | **q-value** | **Pathway**  **source** |
|  | ***Extracellular matrix organization*** | ***293*** | ***118 (40.3%)*** | ***1.81E-08*** | ***6.55E-05*** | ***Reactome*** |
|  | ***Integrin*** | ***124*** | ***59 (47.6%)*** | ***9.41E-08*** | ***1.70E-04*** | ***INOH*** |
|  | ***ECM-receptor interaction - Homo sapiens*** | ***82*** | ***43 (52.4%)*** | ***1.70E-07*** | ***2.05E-04*** | ***KEGG*** |
|  | ***Collagen chain trimerization*** | ***44*** | ***27 (61.4%)*** | ***5.55E-07*** | ***5.01E-04*** | ***Reactome*** |
|  | Degradation of the extracellular matrix | 105 | 47 (44.8%) | 1.47E-05 | 5.92E-03 | Reactome |
|  | Focal Adhesion | 207 | 81 (39.1%) | 1.10E-05 | 5.92E-03 | Wikipathways |
|  | ***Focal adhesion - Homo sapiens*** | ***199*** | ***78 (39.2%)*** | ***1.48E-05*** | ***5.92E-03*** | ***KEGG*** |
|  | Regulation of Glucokinase by Glucokinase Regulatory Protein | 30 | 19 (63.3%) | 1.42E-05 | 5.92E-03 | Reactome |
|  | ***Vesicle-mediated transport*** | ***620*** | ***205 (33.1%)*** | ***1.18E-05*** | ***5.92E-03*** | ***Reactome*** |
|  | ***Axon guidance*** | ***357*** | ***126 (35.4%)*** | ***1.97E-05*** | ***5.97E-03*** | ***Reactome*** |
|  | Influenza Life Cycle | 53 | 28 (52.8%) | 1.98E-05 | 5.97E-03 | Reactome |
|  | Transport of Mature mRNA Derived from an Intronless Transcript | 40 | 23 (57.5%) | 1.78E-05 | 5.97E-03 | Reactome |
|  | Semaphorin interactions | 64 | 32 (50.0%) | 2.27E-05 | 6.32E-03 | Reactome |
|  | Export of Viral Ribonucleoproteins from Nucleus | 31 | 19 (61.3%) | 2.79E-05 | 6.94E-03 | Reactome |
|  | ***Protein digestion and absorption - Homo sapiens*** | ***90*** | ***41 (45.6%)*** | ***3.05E-05*** | ***6.94E-03*** | ***KEGG*** |
|  | Transport of Mature mRNAs Derived from Intronless Transcripts | 41 | 23 (56.1%) | 3.08E-05 | 6.94E-03 | Reactome |
|  | Membrane Trafficking | 582 | 191 (32.8%) | 3.84E-05 | 8.16E-03 | Reactome |
|  | Arf6 trafficking events | 47 | 25 (53.2%) | 4.72E-05 | 8.97E-03 | PID |
|  | Influenza Infection | 63 | 31 (49.2%) | 4.53E-05 | 8.97E-03 | Reactome |
|  | ***Beta1 integrin cell surface interactions*** | ***66*** | ***32 (48.5%)*** | ***4.98E-05*** | ***8.98E-03*** | ***PID*** |
|  | rho cell motility signaling pathway | 34 | 19 (59.4%) | 5.22E-05 | 8.98E-03 | BioCarta |
|  | Arf6 signaling events | 35 | 20 (57.1%) | 7.10E-05 | 1.04E-02 | PID |
|  | Integrin cell surface interactions | 67 | 32 (47.8%) | 7.21E-05 | 1.04E-02 | Reactome |
|  | NEP/NS2 Interacts with the Cellular Export Machinery | 30 | 18 (60.0%) | 6.79E-05 | 1.04E-02 | Reactome |
|  | Transport of Ribonucleoproteins into the Host Nucleus | 30 | 18 (60.0%) | 6.79E-05 | 1.04E-02 | Reactome |
|  | Aminosugars metabolism | 51 | 26 (51.0%) | 8.63E-05 | 1.20E-02 | EHMN |
|  | Rev-mediated nuclear export of HIV RNA | 33 | 19 (57.6%) | 9.36E-05 | 1.21E-02 | Reactome |
|  | Transport of the SLBP independent Mature mRNA | 33 | 19 (57.6%) | 9.36E-05 | 1.21E-02 | Reactome |
|  | ***Collagen formation*** | ***91*** | ***40 (44.0%)*** | ***1.02E-04*** | ***1.22E-02*** | ***Reactome*** |
|  | Phosphatidylinositol phosphate metabolism | 91 | 40 (44.0%) | 1.02E-04 | 1.22E-02 | EHMN |
|  | Asparagine N-linked glycosylation | 286 | 101 (35.4%) | 1.20E-04 | 1.40E-02 | Reactome |
|  | Influenza Viral RNA Transcription and Replication | 44 | 23 (52.3%) | 1.34E-04 | 1.51E-02 | Reactome |
|  | Disease | 509 | 166 (32.7%) | 1.39E-04 | 1.52E-02 | Reactome |
|  | Estrogen Metabolism Pathway | 15 | 11 (73.3%) | 1.43E-04 | 1.52E-02 | PharmGKB |
|  | Intracellular signaling by second messengers | 244 | 88 (36.1%) | 1.61E-04 | 1.54E-02 | Reactome |
|  | Nuclear Pore Complex (NPC) Disassembly | 34 | 19 (55.9%) | 1.61E-04 | 1.54E-02 | Reactome |
|  | Pentose phosphate pathway | 29 | 17 (58.6%) | 1.62E-04 | 1.54E-02 | EHMN |
|  | Transport of the SLBP Dependant Mature mRNA | 34 | 19 (55.9%) | 1.61E-04 | 1.54E-02 | Reactome |
|  | Viral Messenger RNA Synthesis | 42 | 22 (52.4%) | 1.80E-04 | 1.66E-02 | Reactome |
|  | ***Collagen biosynthesis and modifying enzymes*** | ***67*** | ***31 (46.3%)*** | ***1.94E-04*** | ***1.75E-02*** | ***Reactome*** |
|  | DAG and IP3 signaling | 32 | 18 (56.2%) | 2.15E-04 | 1.75E-02 | Reactome |
|  | Effects of PIP2 hydrolysis | 27 | 16 (59.3%) | 2.12E-04 | 1.75E-02 | Reactome |
|  | Interactions of Rev with host cellular proteins | 37 | 20 (54.1%) | 2.01E-04 | 1.75E-02 | Reactome |
|  | Transport to the Golgi and subsequent modification | 168 | 64 (38.1%) | 2.18E-04 | 1.75E-02 | Reactome |
|  | Vpr-mediated nuclear import of PICs | 32 | 18 (56.2%) | 2.15E-04 | 1.75E-02 | Reactome |
|  | ER to Golgi Anterograde Transport | 137 | 54 (39.4%) | 2.44E-04 | 1.92E-02 | Reactome |
|  | Interactions of Vpr with host cellular proteins | 35 | 19 (54.3%) | 2.69E-04 | 2.07E-02 | Reactome |
|  | Thyroid hormone synthesis - Homo sapiens | 74 | 33 (44.6%) | 2.88E-04 | 2.17E-02 | KEGG |
|  | COPI-mediated anterograde transport | 83 | 36 (43.4%) | 3.05E-04 | 2.20E-02 | Reactome |
|  | Neurexins and neuroligins | 57 | 27 (47.4%) | 3.07E-04 | 2.20E-02 | Reactome |
|  | Pyrimidine metabolism | 136 | 53 (39.3%) | 3.11E-04 | 2.20E-02 | EHMN |
|  | Regulation of RhoA activity | 48 | 23 (50.0%) | 3.16E-04 | 2.20E-02 | PID |
|  | Integrins in angiogenesis | 63 | 29 (46.0%) | 3.44E-04 | 2.34E-02 | PID |
|  | integrin signaling pathway | 33 | 18 (54.5%) | 3.60E-04 | 2.37E-02 | BioCarta |
|  | Laminin interactions | 23 | 14 (60.9%) | 3.61E-04 | 2.37E-02 | Reactome |
|  | Calcium signaling pathway - Homo sapiens | 182 | 67 (37.0%) | 4.08E-04 | 2.41E-02 | KEGG |
|  | Ebola Virus Pathway on Host | 81 | 35 (43.2%) | 4.01E-04 | 2.41E-02 | Wikipathways |
|  | Glucose metabolism | 91 | 38 (42.2%) | 4.05E-04 | 2.41E-02 | Reactome |
|  | Metabolism of non-coding RNA | 52 | 25 (48.1%) | 3.82E-04 | 2.41E-02 | Reactome |
|  | snRNP Assembly | 52 | 25 (48.1%) | 3.82E-04 | 2.41E-02 | Reactome |
|  | lectin induced complement pathway | 12 | 9 (75.0%) | 4.68E-04 | 2.73E-02 | BioCarta |
|  | N-Glycan biosynthesis | 42 | 21 (50.0%) | 5.71E-04 | 3.28E-02 | EHMN |
|  | Nuclear import of Rev protein | 34 | 18 (52.9%) | 5.83E-04 | 3.29E-02 | Reactome |
|  | ***Transport of small molecules*** | ***666*** | ***206 (31.0%)*** | ***6.83E-04*** | ***3.74E-02*** | ***Reactome*** |
|  | VEGFA-VEGFR2 Pathway | 92 | 38 (41.3%) | 6.78E-04 | 3.74E-02 | Reactome |
|  | Nuclear Envelope Breakdown | 51 | 24 (47.1%) | 7.35E-04 | 3.96E-02 | Reactome |
|  | ***Stimuli-sensing channels*** | ***102*** | ***41 (40.2%)*** | ***8.21E-04*** | ***4.36E-02*** | ***Reactome*** |
|  | Aldosterone synthesis and secretion - Homo sapiens | 96 | 39 (40.6%) | 8.54E-04 | 4.45E-02 | KEGG |
|  | Vitamin D3 (cholecalciferol) metabolism | 27 | 15 (55.6%) | 8.62E-04 | 4.45E-02 | EHMN |
|  |  |  |  |  |  |  |
| **c** | **Pathway name** | **Pathway size** | **Genes**  **contained** | **p-value** | **q-value** | **Pathway**  **source** |
|  | ***Extracellular matrix organization*** | ***293*** | ***165 (56.3%)*** | ***9.41E-12*** | ***3.68E-08*** | ***Reactome*** |
|  | ***Transport of small molecules*** | ***666*** | ***324 (48.8%)*** | ***1.38E-10*** | ***2.69E-07*** | ***Reactome*** |
|  | EGFR1 | 455 | 230 (50.8%) | 8.92E-10 | 1.16E-06 | NetPath |
|  | ***Integrin*** | ***124*** | ***77 (62.1%)*** | ***1.13E-08*** | ***1.11E-05*** | ***INOH*** |
|  | Degradation of the extracellular matrix | 105 | 67 (63.8%) | 2.11E-08 | 1.55E-05 | Reactome |
|  | ***Vesicle-mediated transport*** | ***620*** | ***295 (47.6%)*** | ***2.38E-08*** | ***1.55E-05*** | ***Reactome*** |
|  | Focal Adhesion | 207 | 115 (55.6%) | 3.65E-08 | 2.04E-05 | Wikipathways |
|  | ***Collagen formation*** | ***91*** | ***59 (64.8%)*** | ***6.27E-08*** | ***3.06E-05*** | ***Reactome*** |
|  | ***ECM-receptor interaction - Homo sapiens*** | ***82*** | ***54 (65.9%)*** | ***1.04E-07*** | ***4.50E-05*** | ***KEGG*** |
|  | ***Collagen chain trimerization*** | ***44*** | ***33 (75.0%)*** | ***3.20E-07*** | ***1.25E-04*** | ***Reactome*** |
|  | Membrane Trafficking | 582 | 271 (46.6%) | 9.14E-07 | 2.98E-04 | Reactome |
|  | Transport to the Golgi and subsequent modification | 168 | 93 (55.4%) | 8.92E-07 | 2.98E-04 | Reactome |
|  | ER to Golgi Anterograde Transport | 137 | 78 (56.9%) | 1.54E-06 | 4.63E-04 | Reactome |
|  | Insulin Pathway | 45 | 32 (71.1%) | 3.46E-06 | 9.65E-04 | PID |
|  | ***Focal adhesion - Homo sapiens*** | ***199*** | ***105 (52.8%)*** | ***3.78E-06*** | ***9.86E-04*** | ***KEGG*** |
|  | ***Stimuli-sensing channels*** | ***102*** | ***60 (58.8%)*** | ***5.92E-06*** | ***1.45E-03*** | ***Reactome*** |
|  | Focal Adhesion-PI3K-Akt-mTOR-signaling pathway | 301 | 148 (49.2%) | 9.00E-06 | 2.07E-03 | Wikipathways |
|  | ***Axon guidance*** | ***357*** | ***171 (48.0%)*** | ***1.09E-05*** | ***2.37E-03*** | ***Reactome*** |
|  | tRNA charging | 38 | 27 (71.1%) | 2.08E-05 | 4.29E-03 | HumanCyc |
|  | Platelet activation - Homo sapiens | 123 | 68 (55.3%) | 2.67E-05 | 5.22E-03 | KEGG |
|  | Axon guidance - Homo sapiens | 175 | 91 (52.0%) | 3.43E-05 | 6.38E-03 | KEGG |
|  | COPI-mediated anterograde transport | 83 | 49 (59.0%) | 3.63E-05 | 6.44E-03 | Reactome |
|  | O-glycosylation of TSR domain-containing proteins | 39 | 27 (69.2%) | 4.38E-05 | 7.45E-03 | Reactome |
|  | ***Collagen biosynthesis and modifying enzymes*** | ***67*** | ***41 (61.2%)*** | ***4.88E-05*** | ***7.64E-03*** | ***Reactome*** |
|  | Fc epsilon RI signaling pathway - Homo sapiens | 68 | 41 (61.2%) | 4.88E-05 | 7.64E-03 | KEGG |
|  | Ion channel transport | 179 | 92 (51.4%) | 5.54E-05 | 8.02E-03 | Reactome |
|  | Signaling events mediated by PTP1B | 53 | 34 (64.2%) | 5.43E-05 | 8.02E-03 | PID |
|  | Inflammatory mediator regulation of TRP channels - Homo sapiens | 97 | 55 (56.7%) | 6.04E-05 | 8.42E-03 | KEGG |
|  | IL11 | 23 | 18 (78.3%) | 6.60E-05 | 8.60E-03 | NetPath |
|  | Laminin interactions | 23 | 18 (78.3%) | 6.60E-05 | 8.60E-03 | Reactome |
|  | ***Beta1 integrin cell surface interactions*** | ***66*** | ***40 (60.6%)*** | ***8.20E-05*** | ***1.01E-02*** | ***PID*** |
|  | Regulation of RAC1 activity | 39 | 26 (68.4%) | 8.31E-05 | 1.01E-02 | PID |
|  | Fc-epsilon receptor I signaling in mast cells | 62 | 38 (61.3%) | 8.72E-05 | 1.03E-02 | PID |
|  | Assembly of collagen fibrils and other multimeric structures | 48 | 31 (64.6%) | 9.56E-05 | 1.10E-02 | Reactome |
|  | O-linked glycosylation | 116 | 63 (54.3%) | 1.05E-04 | 1.18E-02 | Reactome |
|  | Signaling by Rho GTPases | 434 | 196 (45.7%) | 1.14E-04 | 1.24E-02 | Reactome |
|  | Type I hemidesmosome assembly | 9 | 9 (100.0%) | 1.30E-04 | 1.37E-02 | Reactome |
|  | EPHA forward signaling | 33 | 23 (69.7%) | 1.39E-04 | 1.43E-02 | PID |
|  | Platelet activation, signaling and aggregation | 260 | 125 (48.1%) | 1.52E-04 | 1.52E-02 | Reactome |
|  | Signaling by PDGF | 53 | 33 (62.3%) | 1.63E-04 | 1.60E-02 | Reactome |
|  | Rho GTPase cycle | 144 | 73 (52.1%) | 1.76E-04 | 1.68E-02 | Reactome |
|  | role of erbb2 in signal transduction and oncology | 28 | 20 (71.4%) | 2.26E-04 | 2.10E-02 | BioCarta |
|  | Other semaphorin interactions | 19 | 15 (78.9%) | 2.37E-04 | 2.15E-02 | Reactome |
|  | ***Protein digestion and absorption - Homo sapiens*** | ***90*** | ***50 (55.6%)*** | ***2.54E-04*** | ***2.24E-02*** | ***KEGG*** |
|  | Signaling by Receptor Tyrosine Kinases | 422 | 191 (45.3%) | 2.58E-04 | 2.24E-02 | Reactome |
|  | SLC-mediated transmembrane transport | 245 | 117 (48.0%) | 2.77E-04 | 2.35E-02 | Reactome |
|  | Fc Epsilon Receptor I Signaling in Mast Cells | 42 | 27 (64.3%) | 3.01E-04 | 2.45E-02 | SMPDB |
|  | tRNA Aminoacylation | 42 | 27 (64.3%) | 3.01E-04 | 2.45E-02 | Reactome |
|  | superpathway of inositol phosphate compounds | 69 | 40 (58.0%) | 3.15E-04 | 2.51E-02 | HumanCyc |
|  | Intra-Golgi and retrograde Golgi-to-ER traffic | 186 | 92 (49.5%) | 3.25E-04 | 2.54E-02 | Reactome |
|  | Alpha6 beta4 integrin-ligand interactions | 11 | 10 (90.9%) | 3.52E-04 | 2.64E-02 | PID |
|  | cxcr4 signaling pathway | 11 | 10 (90.9%) | 3.52E-04 | 2.64E-02 | BioCarta |
|  | Insulin secretion - Homo sapiens | 85 | 47 (55.3%) | 4.45E-04 | 3.28E-02 | KEGG |
|  | Asparagine N-linked glycosylation | 286 | 133 (46.7%) | 4.75E-04 | 3.37E-02 | Reactome |
|  | KitReceptor | 70 | 40 (57.1%) | 4.74E-04 | 3.37E-02 | NetPath |
|  | a6b1 and a6b4 Integrin signaling | 45 | 28 (62.2%) | 5.15E-04 | 3.43E-02 | PID |
|  | GPVI-mediated activation cascade | 33 | 22 (66.7%) | 5.18E-04 | 3.43E-02 | Reactome |
|  | Plasma lipoprotein clearance | 33 | 22 (66.7%) | 5.18E-04 | 3.43E-02 | Reactome |
|  | Regulation of signaling by CBL | 18 | 14 (77.8%) | 5.10E-04 | 3.43E-02 | Reactome |
|  | Arf6 signaling events | 35 | 23 (65.7%) | 5.29E-04 | 3.44E-02 | PID |
|  | Inositol phosphate metabolism - Homo sapiens | 73 | 41 (56.2%) | 6.58E-04 | 4.15E-02 | KEGG |
|  | Primary Focal Segmental Glomerulosclerosis FSGS | 73 | 41 (56.2%) | 6.58E-04 | 4.15E-02 | Wikipathways |
|  | BCR | 129 | 66 (51.2%) | 6.92E-04 | 4.29E-02 | NetPath |
|  | Phosphatidylinositol phosphate metabolism | 91 | 49 (53.8%) | 7.66E-04 | 4.68E-02 | EHMN |
|  | Integrin cell surface interactions | 67 | 38 (56.7%) | 7.94E-04 | 4.77E-02 | Reactome |
|  | 3-phosphoinositide biosynthesis | 28 | 19 (67.9%) | 9.00E-04 | 4.98E-02 | HumanCyc |
|  | ABC transporters - Homo sapiens | 45 | 27 (61.4%) | 8.82E-04 | 4.98E-02 | KEGG |
|  | Adenylate cyclase activating pathway | 10 | 9 (90.0%) | 8.68E-04 | 4.98E-02 | Reactome |
|  | B Cell Receptor Signaling Pathway | 98 | 52 (53.1%) | 8.45E-04 | 4.98E-02 | Wikipathways |
|  | EGF-Core | 105 | 55 (52.4%) | 9.14E-04 | 4.98E-02 | Signalink |
|  | Non-integrin membrane-ECM interactions | 42 | 26 (61.9%) | 9.07E-04 | 4.98E-02 | Reactome |
|  | Regulation of Glucokinase by Glucokinase Regulatory Protein | 30 | 20 (66.7%) | 9.31E-04 | 4.98E-02 | Reactome |
|  | Transport of Mature mRNA Derived from an Intronless Transcript | 40 | 25 (62.5%) | 9.28E-04 | 4.98E-02 | Reactome |

**Supplementary Table S5**. Over-expressed pathways from ConsensusPathDB analyses[^24^](#_ENREF_24) performed in Campora (a) Gioi (b), and Cardile (c). The genes containing variants with a fold increase > 5 or monomorphic in TSI and with a MAF > 0.0223 in each village were selected for the analyses.p-value is calculated according to the hypergeometric test based on the number of genes present in both the pathway-based set and input list of genes. q-values represent the p-values corrected for multiple testing using the false discovery rate method. All the pathways with q-value < 0.05 are listed. The pathways significantly over-represented, in common between Campora, Gioi and Cardile, are highlighted in bold.

| **Pathway name** | **Pathway size** | **Campora** | | | **Gioi** | | | **Cardile** | | |
| --- | --- | --- | --- | --- | --- | --- | --- | --- | --- | --- |
|  |  | **Genes contained** | **p-value** | **q-value** | **Genes contained** | **p-value** | **q-value** | **Genes contained** | **p-value** | **q-value** |
| Axon guidance | 357 | 110 (30.9%) | 1.51E-03 | 0.21 | 79 (22.2%) | 3.22E-03 | 0.17 | 127 (35.7%) | 2.04E-05 | 8.19E-03 |
| Beta1 integrin cell surface interactions | 66 | 25 (37.9%) | 8.03E-03 | 0.30 | 26 (39.4%) | 7.91E-06 | 2.44E-03 | 26 (39.4%) | 1.08E-02 | 0.22 |
| Collagen biosynthesis and modifying enzymes | 67 | 27 (40.3%) | 2.19E-03 | 0.21 | 27 (40.3%) | 3.19E-06 | 1.26E-03 | 29 (43.3%) | 1.41E-03 | 0.12 |
| Collagen chain trimerization | 44 | 20 (45.5%) | 1.45E-03 | 0.21 | 26 (59.1%) | 2.11E-10 | 6.50E-07 | 23 (52.3%) | 1.59E-04 | 3.44E-02 |
| Collagen formation | 91 | 33 (36.3%) | 5.64E-03 | 0.27 | 35 (38.5%) | 4.47E-07 | 3.45E-04 | 43 (47.3%) | 8.39E-06 | 5.06E-03 |
| ECM-receptor interaction - Homo sapiens | 82 | 31 (37.8%) | 3.52E-03 | 0.22 | 31 (37.8%) | 3.07E-06 | 1.26E-03 | 38 (46.3%) | 4.74E-05 | 1.43E-02 |
| Extracellular matrix organization | 293 | 98 (33.4%) | 1.33E-04 | 0.10 | 85 (29.0%) | 5.55E-08 | 8.44E-05 | 124 (42.3%) | 4.17E-10 | 1.51E-06 |
| Focal adhesion - Homo sapiens | 199 | 65 (32.7%) | 3.18E-03 | 0.22 | 56 (28.1%) | 2.63E-05 | 6.77E-03 | 73 (36.7%) | 4.41E-04 | 5.91E-02 |
| Integrin | 124 | 43 (34.7%) | 4.55E-03 | 0.24 | 45 (36.3%) | 8.20E-08 | 8.44E-05 | 56 (45.2%) | 2.35E-06 | 2.13E-03 |
| Protein digestion and absorption - Homo sapiens | 90 | 30 (33.3%) | 2.79E-02 | 0.39 | 33 (36.7%) | 3.28E-06 | 1.26E-03 | 38 (42.2%) | 5.07E-04 | 0.061 |
| Stimuli-sensing channels | 102 | 41 (40.2%) | 1.96E-04 | 0.10 | 27 (26.5%) | 7.44E-03 | 0.24 | 46 (45.1%) | 1.91E-05 | 8.19E-03 |
| Transport of small molecules | 666 | 194 (29.2%) | 8.38E-04 | 0.20 | 139 (20.9%) | 1.51E-03 | 0.12 | 224 (33.7%) | 2.09E-06 | 2.13E-03 |
| Vesicle-mediated transport | 620 | 183 (29.5%) | 6.88E-04 | 0.20 | 147 (23.7%) | 1.83E-06 | 1.13E-03 | 205 (33.1%) | 2.41E-05 | 8.73E-03 |

**Supplementary Table S6.** Sensitivity analysis for the 13 over-expressed pathways from ConsensusPathDB analyses. Variants with a fold increase > 5 or monomorphic in TSI and with a MAF > 0.0223, showing a statistically significant difference in AF respect to TSI (p-value for Fisher exact test < 0.05) were used to select the genes for the analyses.

**Supplementary Figure S1**. LD-based demographic inference for Cilento and TSI populations. The effective population size (Ne), estimated in the NeON R package[^25^](#_ENREF_25), is reported from 5,000 to 30,000 years ago.


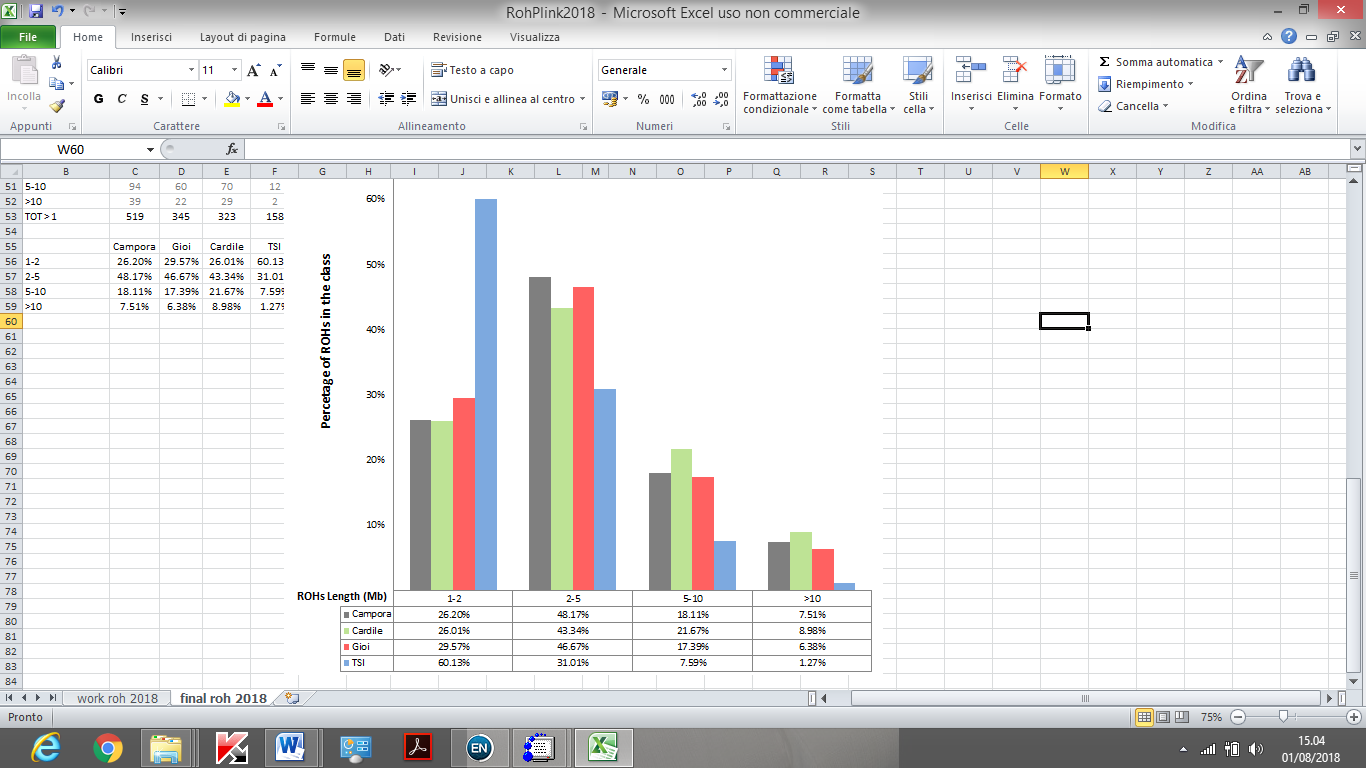


**Supplementary Figure S2.** Distribution of ROHs in Campora, Gioi, Cardile and TSI populations according to their length in Megabases. Only ROHs with length >1 Mb are shown.

**SUPPLEMENTARY REFERENCES**

1 Le Saux, O. *et al.* Mutations in a gene encoding an ABC transporter cause pseudoxanthoma elasticum. *Nat Genet* **25**, 223-227, doi:10.1038/76102 (2000).

2 Le Saux, O. *et al.* A spectrum of ABCC6 mutations is responsible for pseudoxanthoma elasticum. *Am J Hum Genet* **69**, 749-764 (2001).

3 Riazuddin, S. A. *et al.* Missense mutations in TCF8 cause late-onset Fuchs corneal dystrophy and interact with FCD4 on chromosome 9p. *Am J Hum Genet* **86**, 45-53, doi:10.1016/j.ajhg.2009.12.001 (2010).

4 Lechner, J. *et al.* Enrichment of pathogenic alleles in the brittle cornea gene, ZNF469, in keratoconus. *Hum Mol Genet* **23**, 5527-5535, doi:10.1093/hmg/ddu253 (2014).

5 Pasutto, F. *et al.* Mutations in STRA6 cause a broad spectrum of malformations including anophthalmia, congenital heart defects, diaphragmatic hernia, alveolar capillary dysplasia, lung hypoplasia, and mental retardation. *Am J Hum Genet* **80**, 550-560, doi:10.1086/512203 (2007).

6 Sohocki, M. M. *et al.* Mutations in a new photoreceptor-pineal gene on 17p cause Leber congenital amaurosis. *Nat Genet* **24**, 79-83, doi:10.1038/71732 (2000).

7 Gerber, S. *et al.* Complete exon-intron structure of the RPGR-interacting protein (RPGRIP1) gene allows the identification of mutations underlying Leber congenital amaurosis. *Eur J Hum Genet* **9**, 561-571, doi:10.1038/sj.ejhg.5200689 (2001).

8 Kott, E. *et al.* Loss-of-function mutations in RSPH1 cause primary ciliary dyskinesia with central-complex and radial-spoke defects. *Am J Hum Genet* **93**, 561-570, doi:10.1016/j.ajhg.2013.07.013 (2013).

9 Sass, J. O. *et al.* 2-Methylbutyryl-coenzyme A dehydrogenase deficiency: functional and molecular studies on a defect in isoleucine catabolism. *Mol Genet Metab* **93**, 30-35, doi:10.1016/j.ymgme.2007.09.002 (2008).

10 Nogueira, C. P. *et al.* Identification of two different point mutations associated with the fluoride-resistant phenotype for human butyrylcholinesterase. *Am J Hum Genet* **51**, 821-828 (1992).

11 Taroni, F. *et al.* Identification of a common mutation in the carnitine palmitoyltransferase II gene in familial recurrent myoglobinuria patients. *Nat Genet* **4**, 314-320, doi:10.1038/ng0793-314 (1993).

12 Rootwelt, H., Brodtkorb, E. & Kvittingen, E. A. Identification of a frequent pseudodeficiency mutation in the fumarylacetoacetase gene, with implications for diagnosis of tyrosinemia type I. *Am J Hum Genet* **55**, 1122-1127 (1994).

13 Bertina, R. M. *et al.* Mutation in blood coagulation factor V associated with resistance to activated protein C. *Nature* **369**, 64-67, doi:10.1038/369064a0 (1994).

14 Renda, M., Piazza, T., Ciaccio, C. & Maggio, A. Delta + 27 homozygosis in a Sicilian family. *Haematologica* **77**, 82-83 (1992).

15 De Angioletti, M. *et al.* Epidemiology of the delta globin alleles in southern Italy shows complex molecular, genetic, and phenotypic features. *Hum Mutat* **20**, 358-367, doi:10.1002/humu.10132 (2002).

16 Gomez Lira, M. *et al.* High frequency of cystic fibrosis transmembrane regulator mutation L997F in patients with recurrent idiopathic pancreatitis and in newborns with hypertrypsinemia. *Am J Hum Genet* **66**, 2013-2014 (2000).

17 Yehia, L. *et al.* Germline Heterozygous Variants in SEC23B Are Associated with Cowden Syndrome and Enriched in Apparently Sporadic Thyroid Cancer. *Am J Hum Genet* **97**, 661-676, doi:10.1016/j.ajhg.2015.10.001 (2015).

18 Liburd, N. *et al.* Novel mutations of MYO15A associated with profound deafness in consanguineous families and moderately severe hearing loss in a patient with Smith-Magenis syndrome. *Hum Genet* **109**, 535-541, doi:10.1007/s004390100604 (2001).

19 Joutel, A. *et al.* Strong clustering and stereotyped nature of Notch3 mutations in CADASIL patients. *Lancet* **350**, 1511-1515, doi:10.1016/S0140-6736(97)08083-5 (1997).

20 Lee, H. *et al.* Clinical exome sequencing for genetic identification of rare Mendelian disorders. *JAMA* **312**, 1880-1887, doi:10.1001/jama.2014.14604 (2014).

21 Burillo-Sanz, S. *et al.* Mutational profile of rare variants in inflammasome-related genes in Behcet disease: A Next Generation Sequencing approach. *Sci Rep* **7**, 8453, doi:10.1038/s41598-017-09164-7 (2017).

22 Bohring, A. *et al.* WNT10A mutations are a frequent cause of a broad spectrum of ectodermal dysplasias with sex-biased manifestation pattern in heterozygotes. *Am J Hum Genet* **85**, 97-105, doi:10.1016/j.ajhg.2009.06.001 (2009).

23 Zhang, Q. *et al.* Nesprin-1 and -2 are involved in the pathogenesis of Emery Dreifuss muscular dystrophy and are critical for nuclear envelope integrity. *Hum Mol Genet* **16**, 2816-2833, doi:10.1093/hmg/ddm238 (2007).

24 Kamburov, A., Wierling, C., Lehrach, H. & Herwig, R. ConsensusPathDB--a database for integrating human functional interaction networks. *Nucleic Acids Res* **37**, D623-628, doi:10.1093/nar/gkn698 (2009).

25 Mezzavilla, M. G., S. Neon: An R package to estimate human effective population size and divergence time from patterns of linkage disequilibrium between SNPs. *J Comput Sci Syst Biol* **8**, 37-44, doi:10.4172/jcsb.1000168 (2015).
